# Measuring the Level and Potential of China’s Food Security Resilience in the Context of Climate Change

**DOI:** 10.3390/foods15183314

**Published:** 2026-09-19

**Authors:** Lunzheng Zhou, Runzhi Wang

**Affiliations:** 1 School of Economics and Management, East China Jiaotong University, Nanchang 330013, China; 2 School of International Studies, East China Jiaotong University, Nanchang 330013, China; 18770089192@163.com

**Keywords:** food security resilience, climate change, entropy–TOPSIS method, potential forecasting

## Abstract

Intensifying global climate change poses a severe threat to agricultural production, and strengthening China’s food security resilience is therefore of great practical significance for safeguarding food security in China. This study constructs an evaluation system comprising 17 indicators across four dimensions—supply availability, consumption access, food utilization, and system stability—and applies the entropy–TOPSIS method, the Dagum Gini coefficient, kernel density estimation, Markov chains, an obstacle degree model, and the GM(1,1) grey prediction model to measure the level and potential of food security resilience across 31 provinces in China. The results show that China’s food security resilience exhibits a steady upward trend, with the composite score rising from 0.2240 in 2010 to 0.3504 in 2024. Regional disparities are dominated by inter-group differences, whose average contribution rate reaches 70.13%, and the overall Gini coefficient declines gradually. Resilience shows a significant positive spatial autocorrelation throughout, with the global Moran’s I remaining significant at the 1% level in all years. Transitions among resilience levels are characterized by “club convergence”, with the high-level club being the most stable, and neighborhood states significantly condition local transitions. The grain self-sufficiency rate, rural broadband access per capita, and the soil erosion control rate are the leading obstacle factors; at the dimension level, the obstacle degree of supply availability resilience rises and ranks highest by 2024. Panel econometric results further confirm that flood shocks and temperature variability significantly undermine food security resilience, providing direct evidence for the climate change context. Forecasts indicate that national food security resilience will rise to around 0.42 by 2029, with the eastern region maintaining its lead; Tianjin and Beijing rank at the top, while Tibet is the only province projected to decline. The findings of this study provide a reference for the Chinese government in formulating policies to enhance food security resilience and offer a Chinese solution for promoting global food security.

## 1. Introduction

Climate change has become one of the most severe non-traditional security challenges facing human society today. The Sixth Assessment Report of the IPCC indicates that global surface temperatures continue to rise and that extreme heat, drought, rainstorm, and flood events occur with increasing frequency and recurrence, posing systemic threats to agricultural production and food security [1]. Located in the East Asian monsoon region, China has agricultural production that is highly dependent on climatic conditions, making it one of the areas most sensitive to climate change. Extreme weather has already caused significant reductions in the output of China’s major grain crops such as wheat and rice. In 2025, the crop area affected by meteorological disasters nationwide reached 6069.4 thousand hectares, with direct economic losses of 241.617 billion yuan, and the challenges confronting China’s food security in the context of climate change are becoming increasingly protracted and complex [2]. The No. 1 Central Document issued by China in 2026—the Opinions of the CPC Central Committee and the State Council on Anchoring Agricultural and Rural Modernization and Solidly Advancing Comprehensive Rural Revitalization—calls for strengthening the agricultural disaster prevention and mitigation system and improving the capacity to cope with extreme weather [3]. How to enhance food security resilience in the context of climate change has thus become a pressing practical question that demands an answer.

Food security, food system capacity, vulnerability, and resilience are related but distinct concepts. Following the FAO definition, food security is essentially a static performance outcome at a given point in time [4]; food system capacity refers to the resource endowments supporting this outcome, and vulnerability captures the degree to which the system is susceptible to climate shocks. Resilience, by contrast, is a dynamic system property: since Holling [5] introduced the concept into ecosystem research, it has been extended to agricultural and food systems [6,7], denoting the capacity to resist and absorb shocks, maintain core functions during disturbances, and recover—or even transform and upgrade—afterwards. High food security performance therefore does not necessarily imply strong resilience. Because directly tracing realized recovery processes would require high-frequency post-disaster microdata unavailable at the provincial scale, this study follows the mainstream evaluation literature [6,7,8] in operationalizing resilience as the system’s measurable capacity endowments, and the panel regression in Section 3.1 verifies that the resulting scores respond significantly to realized climate shocks, lending ex post empirical support to this operationalization. Enhancing food security resilience in the context of climate change is both an inherent requirement for safeguarding national food security and an inevitable choice for building up China’s strength in agriculture. However, existing research has yet to provide systematic and coherent quantitative answers to a series of questions: what level has China’s food security resilience reached, what spatiotemporal patterns does it exhibit, what factors drive it, what weaknesses constrain it, and how much potential is there for future improvement? These questions constitute the starting point of this study.

Existing research on food security resilience and its relationship with climate change is abundant. Overall, the relevant studies have unfolded along three main lines: research frameworks, evaluation systems, and potential forecasting.

First, in terms of research frameworks, Zuo and Ye [9] and Li et al. [8] constructed evaluation frameworks for food system resilience and agricultural economic resilience along capability dimensions such as risk resistance, adaptive adjustment, and innovative transformation; Yin et al. [10], Zhang et al. [11], and Yang et al. [12] built specialized frameworks for the climate resilience of grain production, supply chain resilience, and food security system transformation; and Xie et al. [13], Tasnim et al. [14], and Li et al. [15] conducted multidimensional or configurational evaluations using multidimensional indicators, household assets, and the entropy-weighted TOPSIS method. Liao et al. [16], Yang and Chen [17], Han et al. [18], Luo et al. [19], Chen et al. [20], and Lin et al. [21] further examined spatial spillovers, spatiotemporal evolution, and obstacle factor diagnosis. Overall, however, most of these frameworks address only a single step—resilience measurement, disparity decomposition, spatiotemporal evolution, or causal diagnosis—and the models underlying each step are mutually independent with weak logical links. A progressive, multi-model integrated analytical framework that interlocks “level measurement–disparity decomposition–spatiotemporal evolution–obstacle diagnosis–potential forecasting” remains rare.

Second, in terms of evaluation systems, although indicator systems have been continuously enriched, the selection of indicators is only loosely connected to the transmission chain of climate shocks, making it difficult to fully reflect the compound risks facing food systems in the context of climate change. Existing evaluations have largely focused on conventional grain production factors and macro-level system performance: some emphasize production and circulation stages such as grain production capacity, yield per unit area, and supply chain efficiency [9,10,11]; some focus on consumption and utilization stages such as dietary structure, household assets, and farmer welfare [12,14]; and others approach the macro-level resistance, recovery, and transformation capacities of the agricultural economic system and supply chains [8,21,22]. Attempts to incorporate climatic elements, such as those of Yin et al. [10] and Xie et al. [13], either characterize climate resilience through production performance under disasters or center on a single stage, without systematically including climate-adaptive indicators such as disaster prevention and mitigation, ecological adaptation, and risk sharing. Measurement frameworks organized around climate risks remain inadequate, making it difficult to characterize the true level of food security resilience in the context of climate change.

Third, in terms of potential forecasting, most existing studies assess the future trajectory of food security qualitatively: Wang et al. [23] designed production scenarios for China’s soybean potential, Wen et al. [24] and Du and Gong [25] explored policy pathways for grain production capacity enhancement, and Fan et al. [26] and Zhang and Er [27] outlined policy directions for strengthening food system resilience from a risk-prevention perspective. In terms of quantitative forecasting, Xiong et al. [28], Gao et al. [29], and Wang et al. [30] projected cereal output, spatiotemporal production changes, and supply–demand balances under coupled climate and socio-economic scenarios, but these forecasts have mostly stopped at single-dimensional projections of output or production capacity; few have incorporated development potential into the analytical framework of resilience evolution to quantitatively assess the future room for optimization of China’s food security resilience system under climate change.

Accordingly, taking climate change as the premise, this study integrates the FAO’s four-dimensional definition of food security with the connotation of resilience capacity, constructs an evaluation system for food security resilience in the context of climate change, and conducts a progressive empirical analysis of “level measurement–driving-effect verification–disparity decomposition–spatiotemporal evolution–obstacle diagnosis–potential forecasting” for 31 Chinese provinces from 2010 to 2024. The marginal contributions are reflected in the substantive findings rather than in the accumulation of methods. First, it provides a systematic quantitative account of the level and evolution of China’s food security resilience, revealing pronounced “club convergence” in level transitions and significant neighborhood effects. Second, rather than treating climate change as a mere background setting, it verifies the significant negative driving effects of flood shocks and temperature variability econometrically and identifies the leading obstacle factors and their regional heterogeneity. Third, it applies the GM(1,1) grey prediction model to quantitatively forecast food security resilience for 2025–2029, clarifying the room for future improvement and providing a decision-making reference for differentiated policy design.

## 2. Materials and Methods

### 2.1. Construction of the Indicator System

(1) **Rationale of the system**. According to the classic definition of the FAO, food security encompasses four dimensions: Availability, Access, Utilization, and Stability [4]. Climate change affects all four dimensions comprehensively by altering light, temperature, water, and soil conditions and by intensifying extreme events [31,32]. Synthesizing the resilience thinking of Holling [5], Folke [33], Béné et al. [6], and Tendall et al. [7], this study defines food security resilience in the context of climate change as the capacity of a food system, under the impacts of climate change and the extreme weather events it induces, to absorb and accommodate climate risks, maintain the uninterrupted functioning of its core functions, and achieve recovery and even transformation and upgrading through rapid response, flexible reorganization, and resource optimization.

(2) **Architecture of the system**. Taking the FAO’s four-dimensional definition as the first-level indicators and embedding the connotation of resilience capacity into each dimension, this study constructs an evaluation system comprising four first-level indicators—supply availability resilience, consumption access resilience, food utilization resilience, and system stability resilience—together with 8 second-level indicators and 17 third-level indicators (Table 1). Supply availability resilience covers production and supply capacity and climate resistance capacity; consumption access resilience covers economic access capacity and market circulation capacity; food utilization resilience covers nutritional outcomes and safe utilization conditions; and system stability resilience covers climate disaster buffering and protection and risk sharing and adjustment. Mapped onto the capacity chain of resilience, the indicator system embodies the four capacities of resistance, absorption, recovery, and adaptation–transformation: the production and supply capacity and the climate resistance capacity (grain yield per unit area, grain self-sufficiency rate, multiple cropping index, and agricultural mechanization level) capture the system’s resistance, i.e., its ability to withstand climate shocks without losing supply functions; the climate disaster buffering indicators (soil erosion control rate and effective irrigation rate) capture absorptive capacity, attenuating the impact intensity of shocks; the risk sharing and adjustment indicators (agricultural insurance loss ratio, intensity of fiscal support for agriculture, and rural pension insurance participation rate) provide the financial and institutional resources underpinning post-shock recovery; and the economic access and market circulation indicators (per capita disposable income of rural residents, Engel’s coefficient, road network density, and rural broadband access per capita) reflect adaptive and transformative capacity, enabling flexible reorganization and resource reallocation across regions and stages, while the utilization dimension ensures the continuity of nutritional outcomes throughout the shock–recovery process. The entropy weights are relatively balanced across the four first-level dimensions—consumption access (28.86%), supply availability (28.64%), system stability (28.32%), and food utilization (14.18%). Following the IPCC risk framework, climate change is conceptualized as an external shock rather than a constituent of resilience itself: the indicator system therefore captures the system’s capacity endowments—production foundation, climate resistance, disaster buffering, and risk sharing—through which climate shocks are absorbed and buffered, so that each indicator is selected for its climate-relevance while no climatic shock variable is mechanically mixed into the score.

(3) **Indicator validity**. First, per capita grain consumption captures the quantity security of staple food intake, while the consumption of high-quality protein foods captures the quality upgrading of the diet. Second, the agricultural insurance loss ratio is defined as the pressure on the risk sharing system under realized disaster shocks—a higher ratio indicates larger payouts relative to premiums and thus heavier disaster losses—rather than as insurance coverage or protection adequacy, and it is assigned a negative direction on this basis. Third, climatic shock variables are not mixed into the indicator system: following the IPCC risk framework, climate change is treated as an external shock to the food system, whose exposure indicators (e.g., flood-affected rate and temperature variability) enter the panel regression in Section 3.1 as explanatory variables, whereas the indicator system measures the system’s capacity to resist, absorb, and recover from such shocks.

(4) **Robustness of the weighting scheme**. To examine the effectiveness of the indicator system, the baseline entropy-weighted scores are compared with two alternative schemes—equal weights and a Top-3 stress test. The resulting scores are highly correlated with the baseline scores, with Pearson coefficients of 0.908 (*p* < 0.01) and 0.872 (*p* < 0.01), respectively (Table 2): even when the three dominant indicators are effectively neutralized, the ranking of provinces is largely preserved, indicating that the measurement results are not driven by a few high-weight indicators and supporting the robustness of the subsequent analyses.

### 2.2. Data Sources and Treatment of Missing Observations

The data used in this study are drawn from the *China Statistical Yearbook*, *China Rural Statistical Yearbook*, *China Environment Statistical Yearbook*, *China Transport Statistical Yearbook*, *Finance Yearbook of China*, *China Labour Statistical Yearbook*, *China Urban-Rural Construction Statistical Yearbook*, *China Health Statistical Yearbook*, and *China Insurance Yearbook*, as well as the Survey on Household Income, Expenditure and Living Conditions conducted by the National Bureau of Statistics and the statistical yearbooks of individual provinces. The sample is a balanced panel of 31 Chinese provinces (excluding Hong Kong, Macao, and Taiwan) from 2010 to 2024, comprising 465 observations. The third-level indicators are calculated from raw data according to the definitions in Table 1; individual missing values within the sample interval are filled by linear interpolation, while missing values in boundary years are extended using nearest-neighbor values [8,9,13], so as to avoid trend extrapolation amplifying end-point fluctuations.

### 2.3. Combination of Research Methods and the Logical Chain

**Figure 1:**
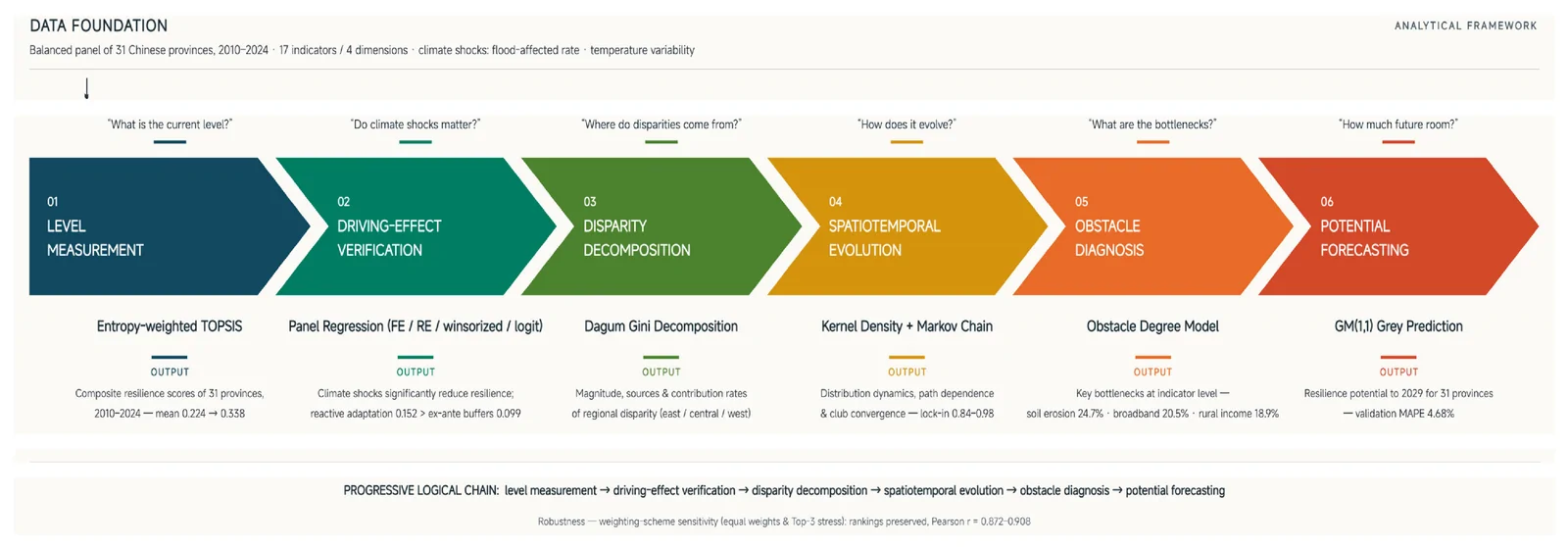
Logical flow chart of the research design

The empirical analysis of this study follows the logical chain of “level measurement–driving-effect verification–disparity decomposition–spatiotemporal evolution–obstacle diagnosis–potential forecasting” (Figure 1). The entropy–TOPSIS method first measures the composite score of food security resilience, answering “what is the current status” and providing the dependent variable for the regression analysis. The panel regression model then tests whether climate change exerts a significant driving effect on resilience, supplying econometric evidence for the “in the context of climate change” premise. On this basis, the Dagum Gini coefficient decomposition identifies “where the disparities lie and where they come from” [8]; kernel density estimation and the (spatial) Markov chain jointly characterize “how the distribution changes and where the system is evolving” [10,16]; the obstacle degree model diagnoses “what the bottlenecks are” at the indicator level [18]; and the GM(1,1) grey prediction model assesses “how the system may evolve in the future” [29]. The robustness of the indicator system underpinning the chain is examined in Section 2.1 through a sensitivity analysis of the weighting scheme.

#### 2.3.1. Entropy–TOPSIS Method

The entropy weight method is an objective weighting approach that determines weights according to the degree of dispersion of indicator data: the greater the dispersion of an indicator, the more information it carries and the higher its weight [8]. TOPSIS (Technique for Order Preference by Similarity to Ideal Solution) ranks evaluation objects by gauging their relative closeness to the positive and negative ideal solutions [15]. This study applies the entropy–TOPSIS method to the evaluation system in Table 1 to calculate the composite scores and dimensional scores of food security resilience for 31 Chinese provinces from 2010 to 2024, answering the level-measurement question of “what is the current status”; the resulting scores also serve as the dependent variable of the panel regression model in Section 2.3.2. To ensure the intertemporal comparability of the scores, all indicators are standardized, the entropy weights are calculated, and the positive and negative ideal solutions are determined on the pooled panel of 465 province-year observations rather than separately for each year, so that the min–max bounds, the weights, and the ideal solutions are common across years and the resulting scores are directly comparable over time. The model is constructed step by step as follows:

Step 1: Construct the initial evaluation matrix. Let *n* denote the number of provinces (evaluation objects) and *m* the number of indicators; the raw indicator data form the following matrix:

(1) $$X\;=\;{\left(x_{ij}\right)}_{n\times m}$$

Step 2: Standardize the indicator values to eliminate differences in dimension and magnitude. For positive indicators:

(2) $${x^{\prime }}_{ij}\;=\;\frac{x_{ij}\;-\;\min\left(x_{j}\right)}{\max\left(x_{j}\right)\;-\;\min\left(x_{j}\right)}$$

For negative indicators:

(3) $${x^{\prime }}_{ij}\;=\;\frac{\max\left(x_{j}\right)\;-\;x_{ij}}{\max\left(x_{j}\right)\;-\;\min\left(x_{j}\right)}$$

Step 3: Calculate the entropy weight of each indicator. First, calculate the proportion of province i under indicator j:

(4) $$p_{ij}\;=\;\frac{{x^{\prime }}_{ij}}{\sum_{i=1}^{n}{x^{\prime }}_{ij}}$$

Second, calculate the information entropy of indicator *j*:

(5) $$e_{j}\;=\;-\frac{1}{\ln n}\sum_{i=1}^{n}p_{ij}\;\ln p_{ij}$$

Finally, calculate the objective weight of indicator *j*:

(6) $$w_{j}\;=\;\frac{1\;-\;e_{j}}{\sum_{j=1}^{m}\left(1\;-\;e_{j}\right)}$$

Step 4: Construct the weighted standardized decision matrix by multiplying the standardized values by the corresponding entropy weights:

(7) $$z_{ij}\;=\;w_{j}\;\times \;{x^{\prime }}_{ij}$$

Step 5: Determine the positive and negative ideal solutions on each indicator:

(8) $$\begin{array}{c} z_{j}^{+}\;=\;\max\left(z_{1j},\;z_{2j},\;\ldots ,\;z_{nj}\right)\\ z_{j}^{-}\;=\;\min\left(z_{1j},\;z_{2j},\;\ldots ,\;z_{nj}\right) \end{array}$$

Step 6: Calculate the Euclidean distances from each evaluation object to the positive and negative ideal solutions:

(9) $$D_{i}^{\pm }\;=\;\sqrt{\sum_{j=1}^{m}{\left(z_{ij}\;-\;z_{j}^{\pm }\right)}^{2}}$$

Step 7: Calculate the relative closeness of each province to the ideal solution, which is taken as the composite score of food security resilience:

(10) $$C_{i}\;=\;\frac{D_{i}^{-}}{D_{i}^{+}\;+\;D_{i}^{-}}$$

where $x_{ij}$ is the raw value of indicator *j* for province *i*; ${x^{\prime }}_{ij}$ is the standardized indicator value; $p_{ij}$ is the proportion of indicator j for province i; $e_{j}$ is the information entropy of indicator j; $w_{j}$ is the objective weight of indicator j; n is the number of provinces and m is the number of indicators; $z_{ij}$ is the weighted standardized value; $z_{j}^{+}$ and $z_{j}^{-}$ are the positive and negative ideal solutions; $D_{i}^{+}$ and $D_{i}^{-}$ are the Euclidean distances between evaluation object i and the positive and negative ideal solutions; and $C_{i}$ is the relative closeness, with larger values indicating a higher level of food security resilience.

#### 2.3.2. Panel Regression Model

To empirically test the driving effects of climate change on food security resilience, this study constructs a panel regression model in which the composite score of food security resilience (C), measured by the entropy–TOPSIS method, serves as the dependent variable. Following the IPCC risk framework, climate change is treated as an external shock to the food system; accordingly, three climate variables are introduced as core explanatory variables—the flood-affected rate (flood-rate, the ratio of flood-affected crop area to total sown area), temperature variability (temp-var), and precipitation variability (precip-var, scaled by 100)—while the urbanization rate (urban), the grain sown area ratio (grain-sow-ratio), the primary industry share (primary ratio), and the logarithm of population density (pop-density) are included as control variables. The model is specified as follows:

(11) $$C_{\mathrm{it}}=\alpha_{0}+\beta_{1}{\mathrm{flood}-\mathrm{rate}}_{\mathrm{it}}+\beta_{2}{\mathrm{temp}-\mathrm{var}}_{\mathrm{it}}+\beta_{3}{\mathrm{precip}-\mathrm{var}}_{\mathrm{it}}+\gamma Z_{\mathrm{it}}+\mu_{i}+\lambda_{t}+\varepsilon_{\mathrm{it}}$$

where *Z* denotes the vector of control variables, *μ* and *λ* denote province and year fixed effects, and *ε* is the error term. The choice among the pooled OLS, fixed effects (FE), and random effects (RE) models is determined by the F test, the BP test, and the Hausman test, and cluster-robust standard errors at the provincial level are used throughout. Climate variables are exogenous to grain production decisions, which mitigates reverse-causality concerns. As a robustness check, the dependent variable is re-estimated after a logit transformation, ln(C/(1−C)), accounting for the bounded nature of the TOPSIS relative closeness.

#### 2.3.3. Dagum Gini Coefficient Decomposition

The Dagum Gini coefficient is a commonly used analytical method for identifying regional disparities and their sources [8]. It can precisely decompose the overall regional disparity G into three components—intra-group differences, inter-group differences, and transvariation density—effectively alleviating the problem of cross-regional sample overlap that traditional Gini coefficients and Theil indices cannot handle. This study applies the Dagum Gini coefficient decomposition to measure and decompose the overall disparity in food security resilience across the four major regions, identifying the magnitude, sources, and respective contribution rates of the disparity, which responds to the need to deconstruct “where the disparities lie and where they come from” and serves as the starting point for understanding the formation mechanism of the spatial pattern of resilience.

(12) $$G=\frac{\sum_{j=1}^{k}\sum_{h=1}^{k}\sum_{i=1}^{n_{j}}\sum_{r=1}^{n_{h}}\left|y_{ji}-y_{hr}\right|}{2n^{2}\bar{y}}$$

(13) $$G=G_{w}+G_{nb}+G_{t}$$

where *G* is the overall Gini coefficient of food security resilience; *k* is the number of regions; *n* is the total number of provinces; *n_j_* and *n_h_* are the numbers of provinces within regions *j* and *h*, respectively; *y_ji_* is the food security resilience level of province *i* in region *j*; *y_hr_* is the food security resilience level of province r in region h; *yˉ* is the national mean of food security resilience; and *G_w_*, *G_nb_*, and *G_t_* denote the contributions of intra-group differences, inter-group (net) differences, and transvariation density, respectively, which satisfy the decomposition identity G = Gw + Gnb + Gt.

#### 2.3.4. Kernel Density Estimation

Kernel density estimation is a non-parametric statistical method that visually presents the distributional characteristics of data through continuous and smooth kernel density curves, reflecting information such as the location, shape, extensibility, and polarization of the distribution of the variable under study, thereby revealing the dynamic evolution of its distribution pattern [10]. This study uses kernel density estimation to characterize, from the time dimension, the continuous evolution of the location, number of peaks, and shape of the food security resilience distributions of the nation and the four major regions, answering whether regional disparities are widening or converging and whether polarization exists, which corresponds to the need to characterize “how the distribution changes”. The kernel density estimator is specified as follows:

(14) $$\hat{f}\left(x\right)=\frac{1}{nh}\sum_{i=1}^{n}K\left(\frac{x-x_{i}}{h}\right)$$

where $\hat{f}(x)$ is the kernel density estimate of the resilience level x; $x_{i}$ is the observed value of the i-th sample; n is the number of observations; h is the bandwidth; and K(⋅) is the kernel function, for which the Gaussian kernel is adopted in this study.

#### 2.3.5. Markov Chain

By constructing a state transition probability matrix, the Markov chain characterizes the direction and probability of transitions of the research object among different level states, and can be used to assess the path dependence and “club convergence” characteristics of level transitions [16]. Building on the traditional Markov chain, the spatial Markov chain introduces a spatial lag operator and incorporates neighborhood states into the transition conditions, thereby remedying the inability of static disparity analysis to capture transition processes and their spatial linkages. Based on the composite scores of food security resilience, this study divides the provinces into four levels—low, medium-low, medium-high, and high—according to the three quartiles (Q1, the median, and Q3) of the pooled distribution of the 465 province-year composite scores, so that the low level covers scores below Q1, the medium-low level covers the interval from Q1 to the median, the medium-high level covers the interval from the median to Q3, and the high level covers scores above Q3. This quartile-based scheme is distribution-driven, guarantees a balanced number of observations in each state, and avoids the arbitrariness of ad hoc thresholds; the number of observed transitions (n) underlying each estimated probability is reported together with the results. The study constructs a traditional Markov transition probability matrix to examine the dynamic evolution of resilience levels; it further constructs a spatial Markov chain to analyze the conditional transition probabilities under different neighborhood resilience levels, answering the assessment question of “where the system is evolving”. The state transition probabilities are calculated as follows:

(15) $$p_{ij}=\frac{n_{ij}}{n_{i}}$$

(16) $$p_{ij|k}=\frac{n_{ij|k}}{n_{i|k}}$$

where $p_{ij}$ denotes the probability that a province transitions from level state i to state j in the following year; $n_{ij}$ is the number of provinces transitioning from state i to state j; $n_{i}$ is the total number of provinces in state i; and $p_{ij|k}$ denotes the conditional transition probability under neighborhood type k in the spatial Markov chain, with $n_{ij|k}$ and $n_{i|k}$ defined analogously.

#### 2.3.6. Obstacle Degree Model

The obstacle degree model is a classic method for diagnosing constraining factors on the basis of comprehensive evaluation. It calculates the obstacle degree of each indicator through the factor contribution degree (indicator weight) and the indicator deviation degree (the gap between the actual value of an indicator and its ideal target), thereby locating the key weaknesses constraining the improvement of the evaluation object [18]. This study applies the obstacle degree model to conduct obstacle diagnosis for indicators at all levels of the food security resilience evaluation system, bringing the attribution results down to the level of specific, actionable indicators, and examines the heterogeneity of the obstacle structure by region, answering the diagnostic question of “what the bottlenecks are”. It should be noted that, by construction, the obstacle degree measures the weighted shortfall of an indicator relative to the sample ideal solution—a descriptive diagnostic of where the system falls short of the frontier—and does not identify causal constraints; in particular, indicators carrying higher entropy weights mechanically tend to exhibit higher obstacle degrees. The diagnosis is therefore used to rank weighted shortcomings rather than to establish causal drivers. The model is constructed as follows:

(17) $$I_{ij}=1-{x^{\prime }}_{ij}$$

(18) $$O_{j}=\frac{F_{j}\times I_{j}}{\sum_{j=1}^{m}F_{j}\times I_{j}}\times 100\%$$

where $I_{ij}$ is the deviation degree of indicator j for province i; ${x^{\prime }}_{ij}$ is the standardized value of indicator j; $F_{j}$ is the factor contribution degree of indicator j, represented by its composite weight $w_{j}$; $O_{j}$ is the obstacle degree of indicator j; and m is the number of indicators. A larger $O_{j}$ indicates a stronger constraining effect of the indicator on resilience improvement.

#### 2.3.7. GM(1,1) Grey Prediction Model

The GM(1,1) grey prediction model is a classic forecasting method for small-sample, short-length series with clear trends. It weakens the randomness of the original series through first-order accumulation generation, characterizes the cumulative development tendency with a first-order differential (whitening) equation, estimates the development coefficient and the grey action quantity by least squares, and produces outward recursive forecasts after accuracy testing with the posterior error ratio and the small error probability, thereby enabling relatively robust assessment of the medium- and short-term evolution path of the variable [29]. Food security resilience exhibits pronounced temporal inertia and trend behavior. Based on the time-series data of food security resilience for 31 provinces from 2010 to 2024, this study constructs GM(1,1) models to forecast the food security resilience levels of the nation and individual provinces for 2025–2029 and to measure the gap between each province and the sample ideal frontier, answering the forward-looking question of “how much room there is in the future”. The model is constructed as follows:

(19) $$\frac{dx^{\left(1\right)}}{dt}+ax^{\left(1\right)}=b$$

where *x*^(1)^ is the first-order accumulated series of the food security resilience score; *a* is the development coefficient, characterizing the growth or decay tendency of the series; and *b* is the grey action quantity, capturing the driving effect of system behavior. Both parameters are estimated by least squares, and model accuracy is examined by the posterior error ratio C and the small error probability P before outward recursive forecasting.

## 3. Results

### 3.1. Driving Effects of Climate Change: Panel Econometric Evidence

Panel regression is first used to examine whether climate change exerts a significant driving effect on food security resilience. The F test (F(30,427) = 46.179, *p* < 0.01) and the BP test (χ^2^(1) = 1681.311, *p* < 0.01) both reject the pooled OLS model. Although the Hausman test (χ^2^(7) = 0.930, *p* = 0.996) statistically favors the random effects (RE) model, the two-way fixed effects (FE) model is adopted as the baseline because year fixed effects absorb nationwide common shocks—such as macro policy shifts and large-scale climate anomalies—that the RE model cannot control, and the FE estimator remains consistent even when individual effects are correlated with the regressors. As shown in Table 3, the coefficients of the flood-affected rate (−0.045, *p* < 0.05) and temperature variability (−0.022, *p* = 0.071) are both negative, whereas precipitation variability shows no significant effect; among the control variables, only the grain sown area ratio (0.116, *p* < 0.01) is significantly positive.

Three robustness checks support these results. Under the RE specification favored by the Hausman test, the flood-affected rate (−0.114, *p* < 0.01) and temperature variability (−0.087, *p* < 0.01) are significantly negative, with magnitudes larger than the baseline estimates; the same holds after winsorizing all continuous variables at the 1st and 99th percentiles (−0.141 and −0.088, both *p* < 0.01) and after a logit transformation of the dependent variable (−0.508 and −0.377, both *p* < 0.01). The negative driving effects of flood shocks and temperature variability on food security resilience are therefore robust to the model specification, extreme-value treatment, and functional form, providing direct econometric evidence for the “in the context of climate change” premise of this study.

To further link the measurement results to climate change, the 31 provinces are divided by their mean flood-affected rate over 2010–2024—the most robustly significant climate variable across all the above model specifications and the most spatially differentiated—into a high-exposure group (16 provinces at or above the median of 0.0383) and a low-exposure group (15 provinces); a parallel grouping by temperature variability yields no significant inter-group difference, which is consistent with the weaker regression coefficients of that variable. The two groups did not differ significantly in their initial levels (0.2376 vs. 0.2096 in 2010, *p* = 0.218), but by 2024 the high-exposure group had pulled significantly ahead (0.3898 vs. 0.3084, *p* = 0.010): its mean cumulative gain (0.1522) significantly exceeds that of the low-exposure group (0.0988; t = 2.649, *p* = 0.013), and the inter-group gap widened steadily from 0.028 in 2010 to 0.081 in 2024. The faster improvement of the high-exposure group is not a low-base artifact, as the initial level is insignificant once controlled for. Together with the significantly negative contemporaneous effects identified above, this pattern points to a shock–response mechanism: climate shocks erode food security resilience in the short run, but sustained exposure induces long-term adaptive capacity building, allowing frequently exposed provinces to catch up and overtake in resilience levels.

### 3.2. Overall Development Level and Temporal Evolution

(5) **Analysis of the weight structure**. The entropy-weighted TOPSIS method is used to assign objective weights to the evaluation system: the greater the dispersion of an indicator, the more information it carries and the higher its weight [15]. This data-driven weighting avoids the subjectivity of expert scoring and objectively reflects the information content of each indicator.

**Figure 2:**
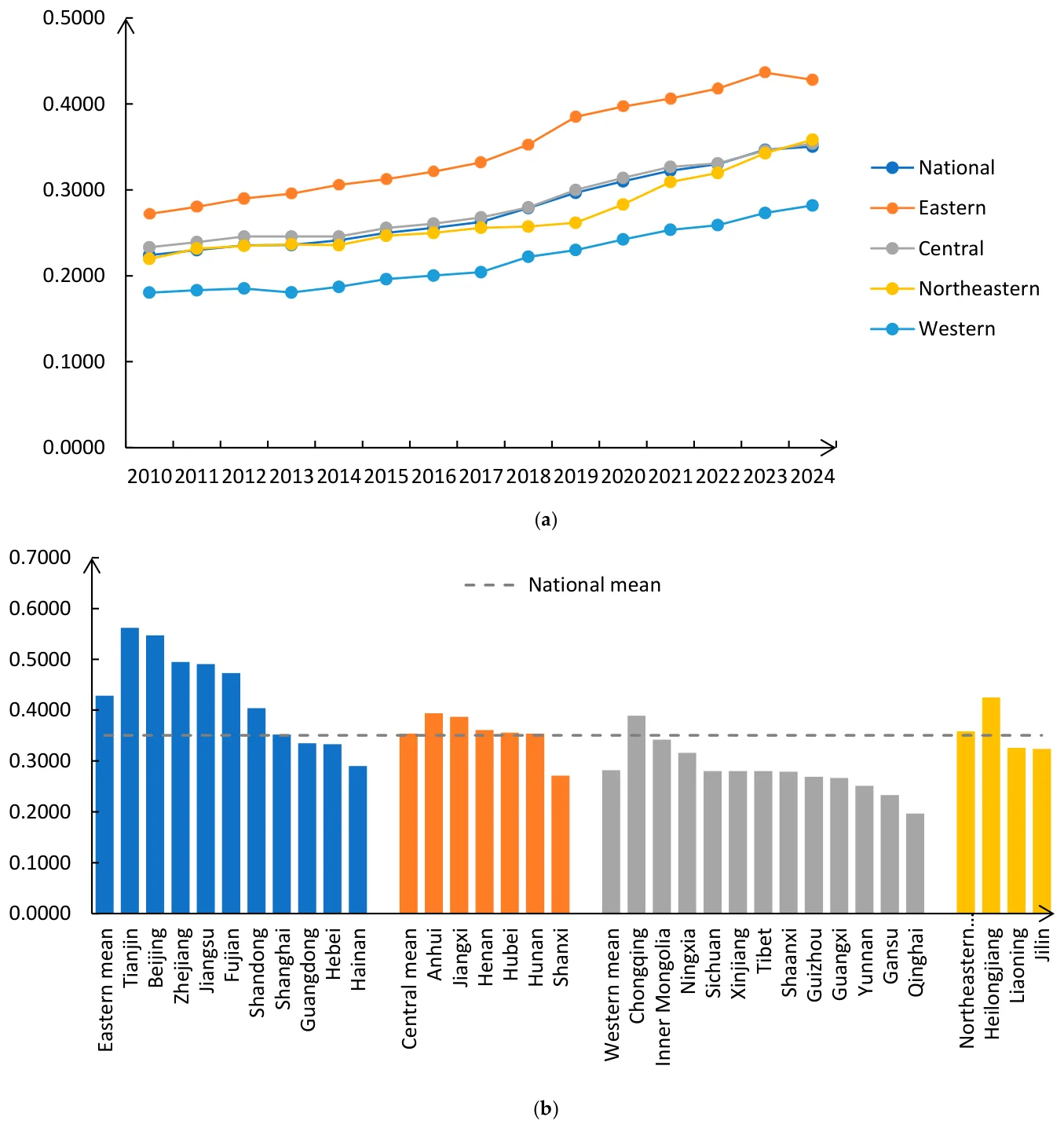
(**a**) Composite scores of food security resilience for the nation and the four major regions, 2010–2024. (**b**) Composite scores of food security resilience for the nation and the four major regions, 2024.

As shown in Figure 2a, China’s food security resilience exhibits a steady and continuous upward trend: the national composite score rose from 0.2240 in 2010 to 0.3504 in 2024, with an average annual growth rate of 3.2475%. The trajectory is smooth throughout, without abnormal jumps or drops. By region, the period averages form a gradient led by the eastern region (0.3484), followed by the central (0.2830), northeastern (0.2696), and western (0.2187) regions. In the 2024 cross-section, the regional means diverged further—to 0.4282 in the eastern, 0.3583 in the northeastern, 0.3538 in the central, and 0.2819 in the western region; the northeastern region, starting from a low base (0.2197 in 2010), grew the fastest and overtook the central region to rank second by 2024.

### 3.3. Spatial Distribution Pattern

**Figure 3:**
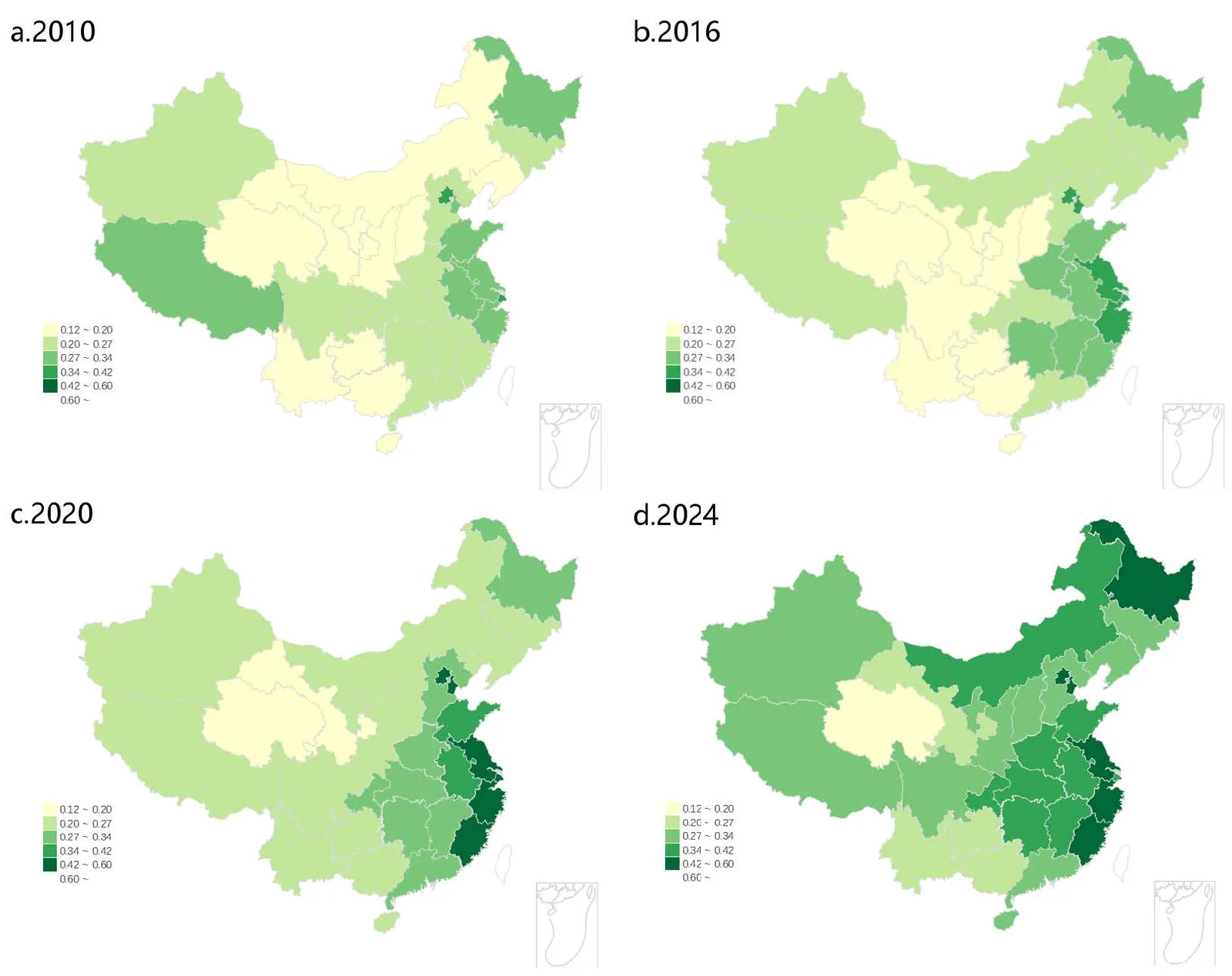
Spatial distribution pattern of China’s food security resilience in 2010, 2016, 2020, and 2024.

According to Figure 3, China’s food security resilience exhibits, overall, a spatial pattern of “high values along the eastern coast, and relatively low values in the western and some central provinces”. In 2024, the eastern coastal provinces and municipalities—Tianjin (0.5620), Beijing (0.5470), Zhejiang (0.4950), Jiangsu (0.4910), and Fujian (0.4730)—formed a high-value belt on the strength of well-developed market circulation and digital infrastructure, higher rural incomes, and solid climate disaster buffering capacity; Qinghai (0.1970), Gansu (0.2330), and Yunnan (0.2510) in the west fell into the low-value area, constrained mainly by weak production and supply foundations and insufficient climate disaster buffering. Compared with 2010, the composite scores of all provinces rose across the board in 2024, with Tianjin, Fujian, Zhejiang, Chongqing, and Jiangsu registering the largest increases. The dominant factors behind inter-provincial divergence lie mainly in market circulation and digital infrastructure, climate disaster buffering, and the production capacity foundation, and the “higher in the east and lower in the west” character of the spatial pattern has become further evident under the new weight structure.

### 3.4. Spatial Correlation Analysis

**Figure 4:**
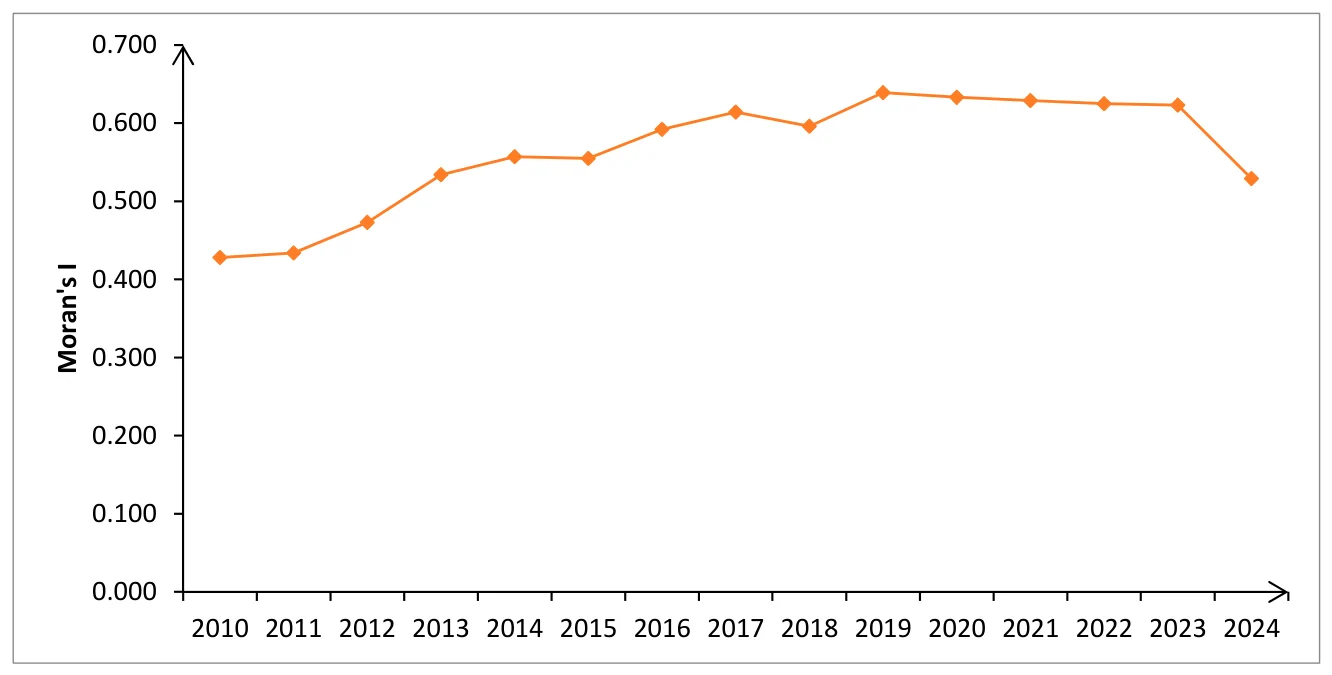
Global Moran’s I index of China’s food security resilience, 2010–2024.

**Figure 5:**
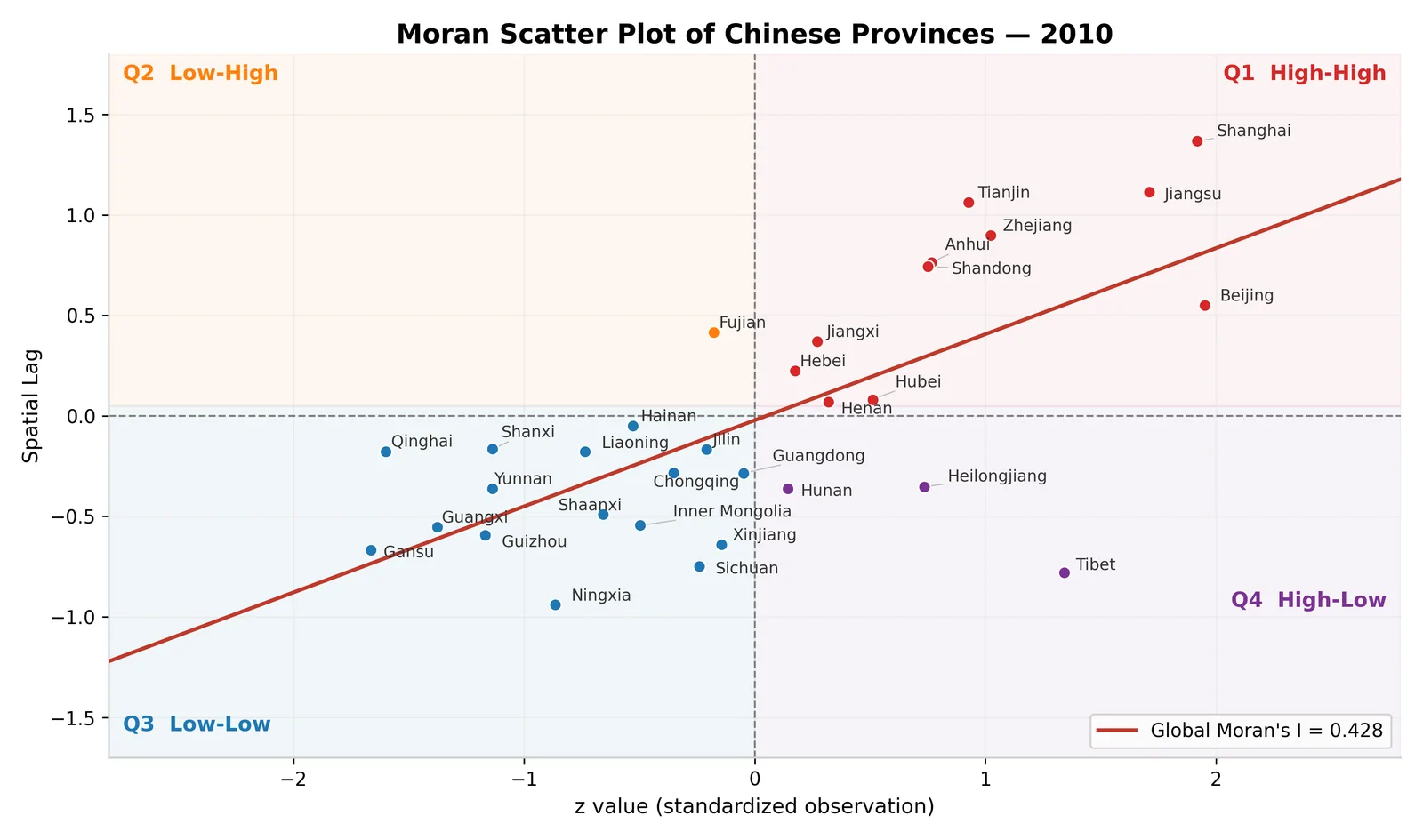

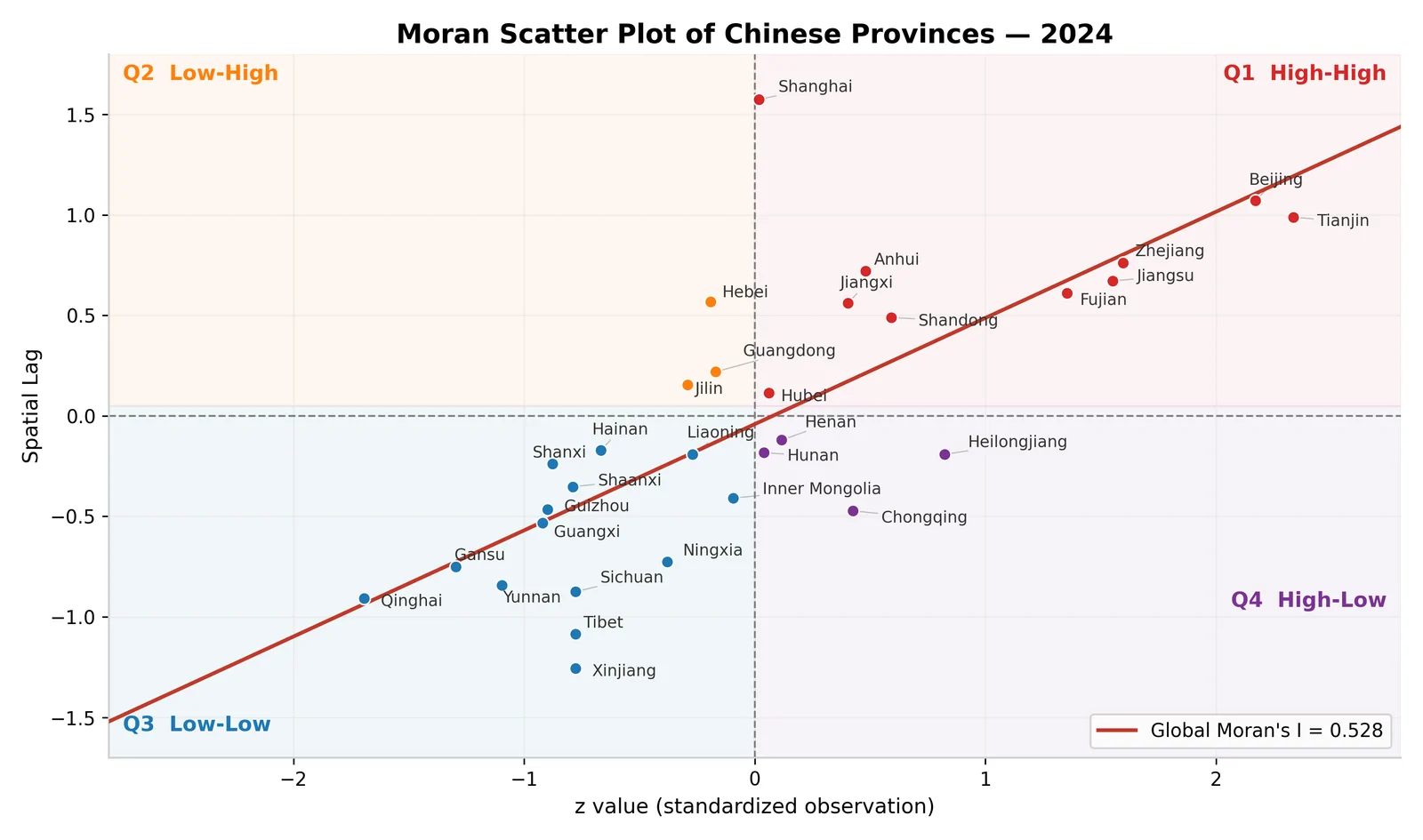
Moran scatterplots of China’s food security resilience in 2010 and 2024.

(1) Global autocorrelation. The global Moran’s I indices are shown in Figure 4. From 2010 to 2024, the global Moran’s I index of China’s food security resilience ranged between 0.4280 and 0.6390, all significantly positive at the 1% level. The index rose from 0.4280 in 2010 to a peak of 0.6390 in 2019, then fluctuated slightly downward to 0.5290 in 2024, remaining at a high level throughout. This indicates a strong and persistent positive spatial correlation in food security resilience among provinces—high-value and low-value provinces each cluster in contiguous areas—with the intensity of agglomeration strengthening markedly during 2010–2019 and easing somewhat thereafter [15].

(2) Local autocorrelation. The Moran scatterplots for 2010 and 2024 are shown in Figure 5. In both years, the majority of provinces are concentrated in quadrants I and III, dominated by “H-H” and “L-L” agglomeration patterns, which is consistent with the significantly positive global Moran’s I throughout the study period and indicates that the local spatial structure of food security resilience is characterized by persistent agglomeration of similar values. In terms of agglomeration composition, the “H-H” agglomeration area is located mainly along the eastern coast, while the “L-L” agglomeration areas are distributed in the western region and parts of the central region; only a few provinces fall in the transitional “L-H” or “H-L” quadrants, and the mismatch between high and low values is limited. It is noteworthy that the “H-H” agglomeration belt largely coincides with the high-exposure provinces identified in Section 3.1—the eastern coastal and central riverside provinces that are most frequently hit by flood shocks are precisely those clustered at high resilience levels—which is consistent with the shock-induced capacity building discussed above. Overall, the spatial dependence whereby high-value and low-value provinces of China’s food security resilience each cluster in contiguous areas is prominent and stable, and the spatial agglomeration pattern has been continuously shaped by the expansion of market circulation, digital infrastructure, and climate disaster buffering capacity.

### 3.5. Regional Disparities in China’s Food Security Resilience

**Figure 6:**
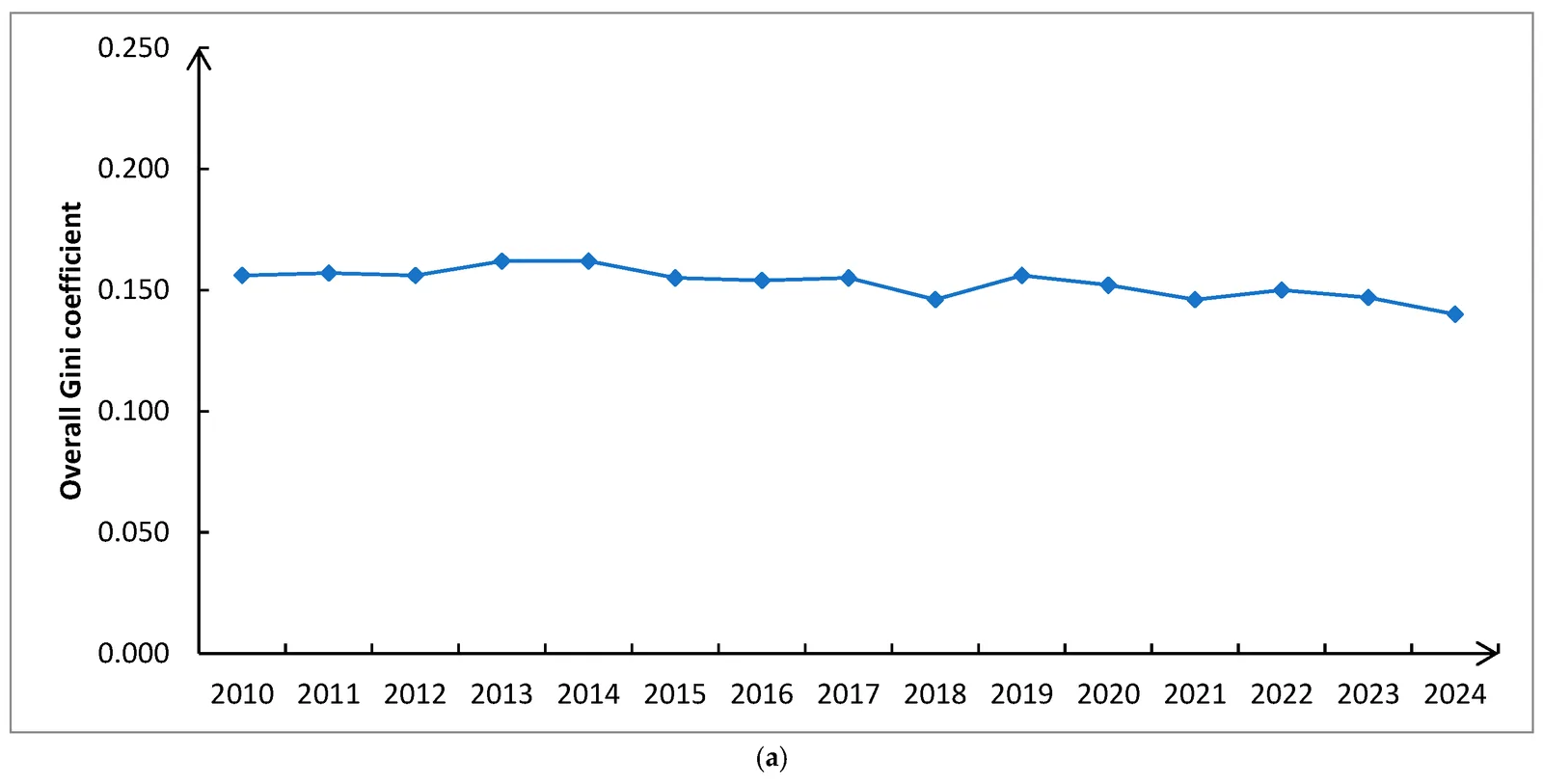

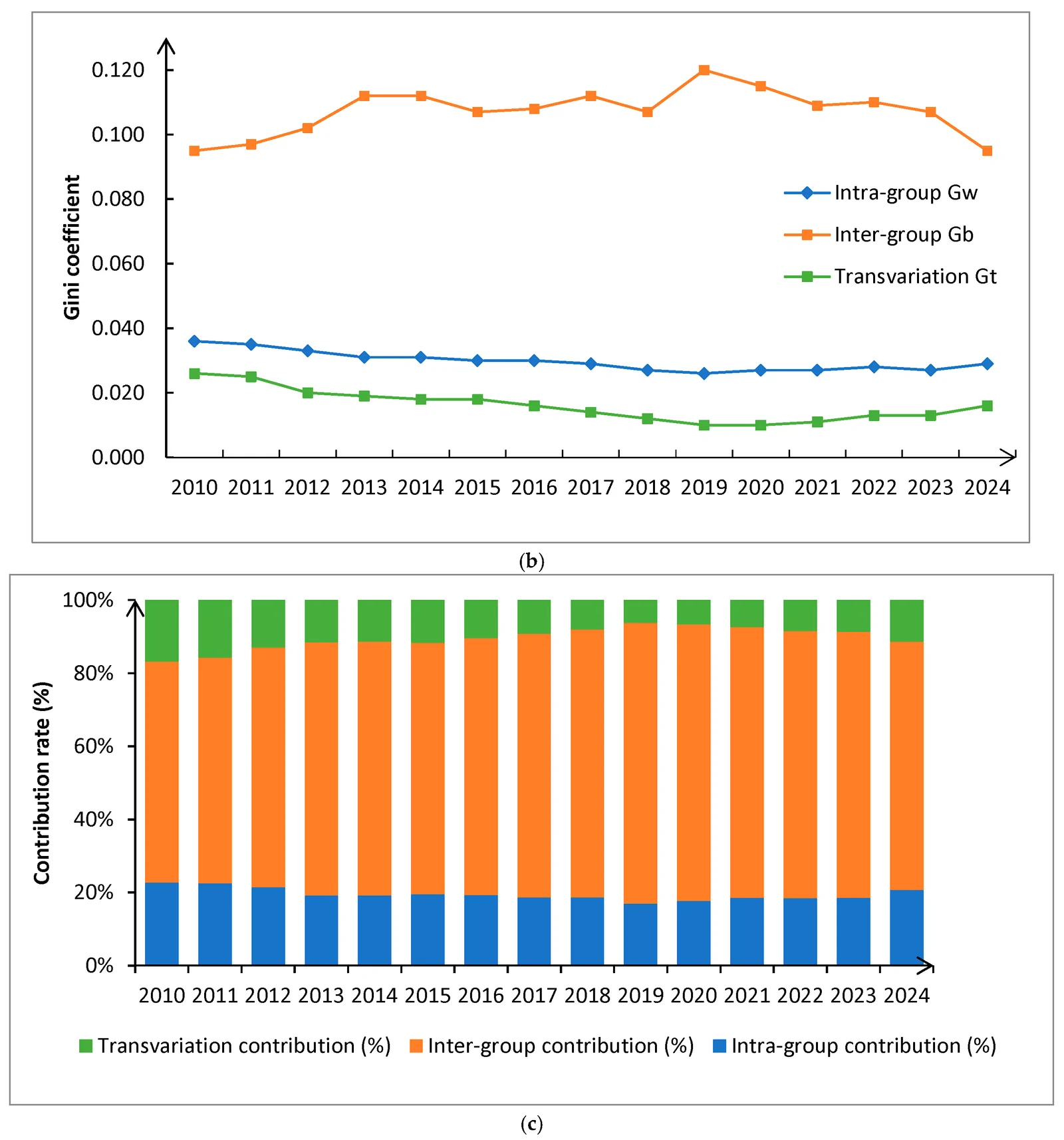
(**a**) Evolution of the overall Gini coefficient of China’s food security resilience, 2010–2024. (**b**) Dagum Gini coefficient decomposition of China’s food security resilience, 2010–2024. (**c**) Contribution rates of the sources of regional disparity in China’s food security resilience, 2010–2024.

According to Figure 6, in terms of overall disparity, the overall Gini coefficient of China’s food security resilience fluctuated within the range of 0.140–0.162 during 2010–2024, rising from 0.156 in 2010 to a peak of 0.162 in 2013–2014 and then declining overall to 0.140 in 2024, exhibiting a generally converging trend (Figure 6a).

The Dagum Gini coefficient decomposition results show that the evolution paths of the three types of disparity diverge markedly. Intra-group differences declined overall from 0.036 in 2010 to 0.029 in 2024, indicating a gradual narrowing of gaps among provinces within regions. Inter-group differences rose from 0.095 in 2010 to a peak of 0.120 in 2019 and then eased back to 0.095 in 2024, returning to the level at the beginning of the study period, indicating that the structural gaps among regional blocs widened first and then converged. This widening of inter-group gaps over most of the period is consistent with the divergence between the high- and low-exposure groups identified in Section 3.1, suggesting that differentiated climate exposure forms part of the background to regional polarization. Transvariation density declined from 0.026 in 2010 to 0.016 in 2024, reflecting a marked weakening of the cross-regional overlap of distributions (Figure 6b).

In terms of disparity sources, inter-group differences are by far the largest contributor, with an average contribution rate of 70.13%, followed by intra-group differences (19.48%), with transvariation density the smallest (10.39%). In terms of trends, the contribution rate of inter-group differences rose from 60.47% in 2010 to a peak of 76.95% in 2019 and remained at 67.99% in 2024, while that of transvariation density fell from 16.80% to 11.27% over the same period; the source structure of regional disparities is thus persistently dominated by structural imbalances between regional blocs (Figure 6c). Therefore, narrowing regional gaps in food security resilience hinges on resolving the structural imbalances between regions—particularly by strengthening the western region’s production foundation, market circulation, and climate disaster buffering capacities—while due attention should also be paid to governing the divergence within regions.

### 3.6. Temporal Evolution: Kernel Density Estimation

**Figure 7:**
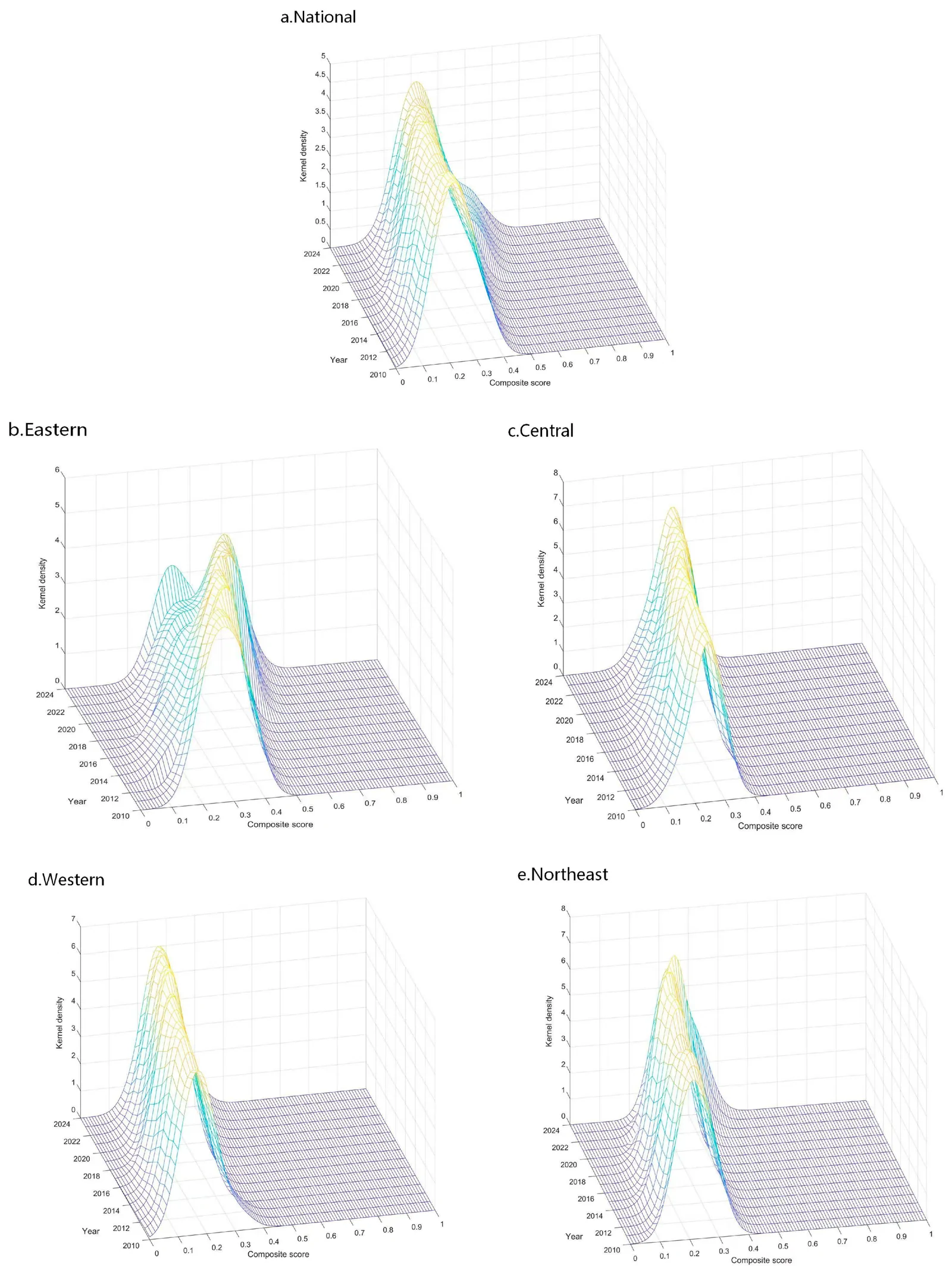
Kernel density distributions of food security resilience in China and its four major regions.

Figure 7 presents the three-dimensional kernel density surfaces of food security resilience for the nation and the four major regions with year, composite score, and estimated density on the three axes, allowing continuous observation of the migration of the distribution center and changes in peak shape and dispersion. At the national level, the surface evolves from a single narrow peak at a low position in 2010 into a rightward-shifted and visibly spread-out form in 2024, with the distribution mean rising from 0.2240 to 0.3504 and the standard deviation widening from 0.0625 to 0.0906; in the later years the peak flattens and the right tail extends, indicating that, alongside the overall improvement, inter-provincial dispersion has intensified—an evolution characterized by “overall upgrading with widening dispersion”.

At the regional level, the main ridges of all four regions migrate toward the high-score interval, but their shapes differ markedly. The eastern surface lies furthest to the right, its mean rising from 0.2721 in 2010 to 0.4282 in 2024, with the peak flattening and the right tail extending—its standard deviation widening from 0.0563 to 0.0976, the largest among the four regions—indicating somewhat widened internal divergence alongside the highest level. The central surface remains a compact single peak, with its mean rising from 0.2332 to 0.3538 and its standard deviation staying low (0.0414 in 2010 and 0.0438 in 2024), exhibiting stable internal convergence. The western surface starts from the lowest position, its mean rising from 0.1805 to 0.2819, and its shape remains dispersed with a gentle peak, indicating persistent intra-regional differentiation at a low level. The northeastern ridge shifts rightward the most, its mean rising from 0.2197 to 0.3583, with the peak remaining relatively concentrated, exhibiting steady upgrading at a moderate level of dispersion.

### 3.7. Dynamic Evolution: Markov Chain Analysis

**Figure 8:**
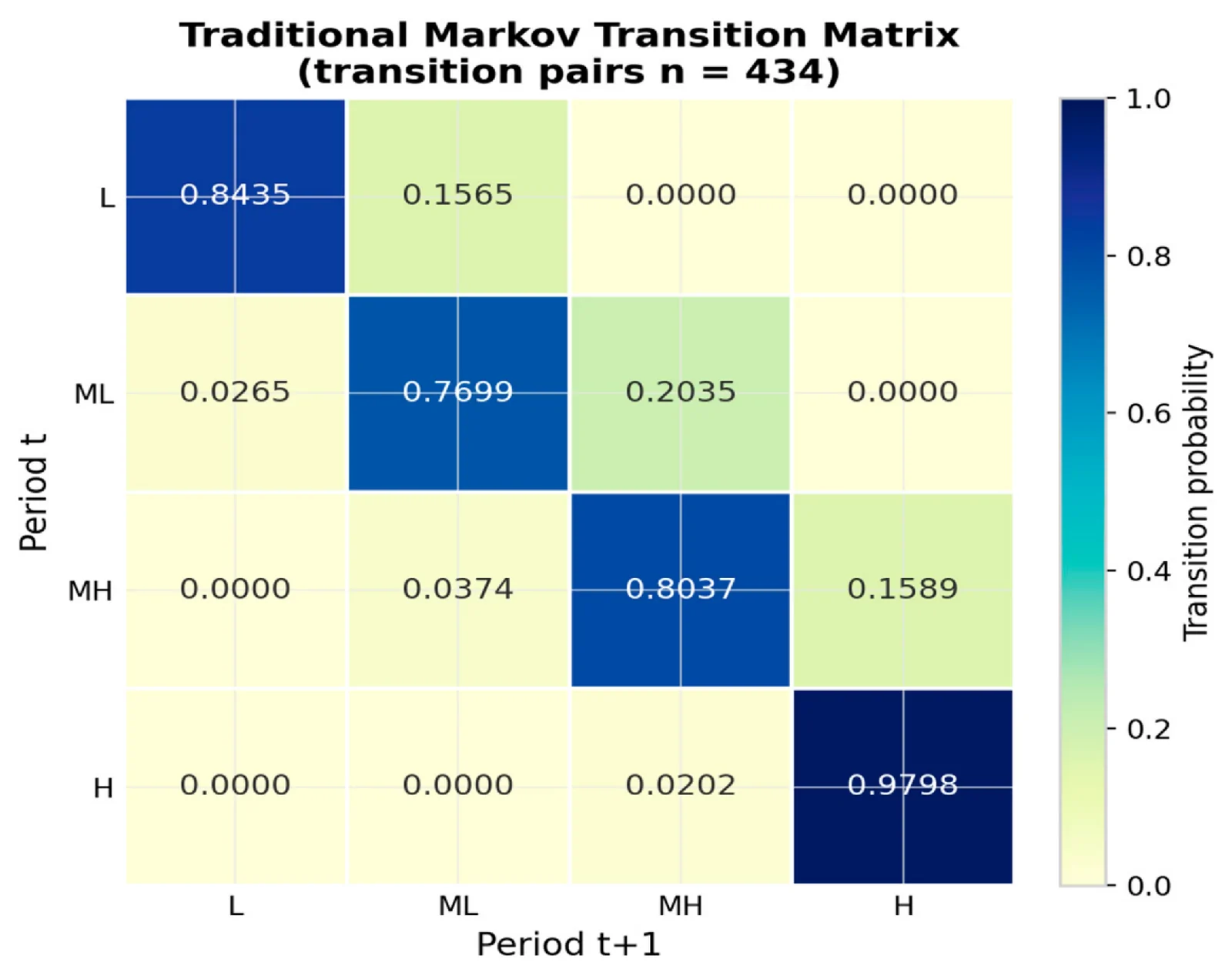
Traditional Markov transition matrix of China’s food security resilience.

The traditional Markov transition probability matrix, estimated from 434 year-to-year transition pairs, is shown in Figure 8. The main-diagonal probabilities (0.8435, 0.7699, 0.8037, and 0.9798, corresponding to 97 of 115, 87 of 113, 86 of 107, and 97 of 99 observed outgoing transitions for the four levels, respectively) far exceed the off-diagonal entries, indicating pronounced state persistence and “club convergence”: high-level provinces are almost fully locked in (0.9798) and low-level provinces are also clearly entrenched (0.8435)—a “Matthew effect” at both ends of the distribution—whereas the medium-low (0.7699) and medium-high (0.8037) levels are relatively more fluid. Notably, seven of the eight provinces in the high-level club in 2024 (Tianjin, Beijing, Zhejiang, Fujian, Heilongjiang, Shandong, and Anhui) belong to the high-exposure group, indicating that once adaptive capacities are built up, frequent climate shocks do not prevent entry into—and stable membership in—the leading club.

Transitions occur exclusively between adjacent levels, with upward momentum clearly dominating: the probabilities of moving one level up from the low, medium-low, and medium-high levels are 15.65%, 20.35%, and 15.89% (18 of 115, 23 of 113, and 17 of 107 transitions, respectively), far exceeding those of moving one level down (2.65%, 3.74%, and 2.02%; 3 of 113, 4 of 107, and 2 of 99, respectively), and cross-level jumps are absent throughout. Resilience improvement is thus a gradual, durable process of stepwise accumulation trending upward overall, closely related to the continuous strengthening of policies to support agriculture and benefit farmers and of disaster prevention, mitigation, and risk protection capacities.

**Figure 9:**
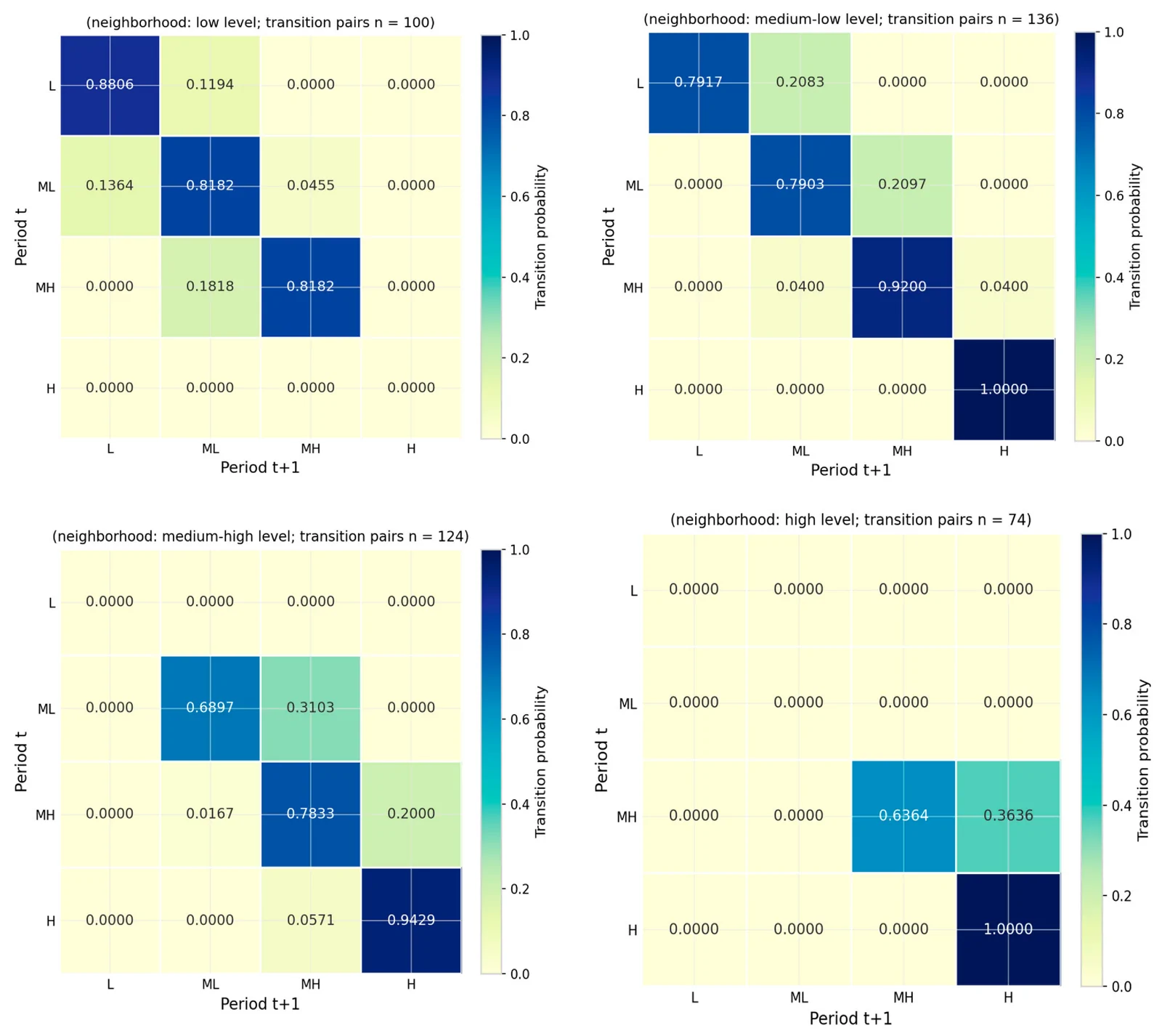
Markov transition probability heatmaps of China’s food security resilience.

The spatial Markov conditional transition matrices under the four neighborhood types are shown in Figure 9 (all-zero rows denote level–neighborhood combinations without observed transitions). Transition probabilities differ markedly across neighborhood types while the diagonal entries remain dominant, indicating that transition patterns are systematically associated with neighborhood states. The lock-in of low-level provinces is highly sensitive to neighborhood states: their state-retention probability reaches 88.06% (59 of 67 transitions) under low-level neighborhoods but falls to 79.17% (38 of 48) under medium-low-level neighborhoods, where the upward probability rises from 11.94% (8 of 67) to 20.83% (10 of 48); no low-level province is observed under medium-high- or high-level neighborhoods, itself reflecting the spatial co-location of similar resilience levels.

An upgrading neighborhood environment is associated with markedly higher upward-transition probabilities: for medium-low-level provinces, the probability of moving up rises from 4.55% (1 of 22) under low-level neighborhoods to 20.97% (13 of 62) and 31.03% (9 of 29) under medium-low- and medium-high-level neighborhoods, respectively; for medium-high-level provinces, the probability of entering the high level rises from 4.00% (1 of 25) under medium-low-level neighborhoods to 20.00% (12 of 60) under medium-high-level neighborhoods and 36.36% (4 of 11) under high-level neighborhoods. Conversely, low-level neighborhoods are associated with downward moves (13.64%, or 3 of 22, of medium-low-level provinces falling to the low level, and 2 of 11 medium-high-level provinces moving downward), and the only observed downgrades of high-level provinces (2 of 35) occur under medium-high-level neighborhoods; the state-retention probability of high-level provinces reaches 100% (63 of 63) and 94.29% (33 of 35) under high- and medium-high-level neighborhoods, respectively, forming a stable high-level club. Overall, an upgrading neighborhood environment is systematically associated with higher upward-transition probabilities and lower downward ones. It should be emphasized that these conditional probabilities are descriptive statistics of observed transitions rather than statistically tested spatial effects: several conditioning rows rest on only a handful of observations (with n as small as 1), so extreme magnitudes such as 100% retention should be read as indicative rather than precise; the statistical significance of spatial dependence itself is established by the Moran’s I tests reported in Section 3.4.

### 3.8. Identifying Weak Links in the Improvement Path: Obstacle Degree Diagnosis

**Figure 10:**
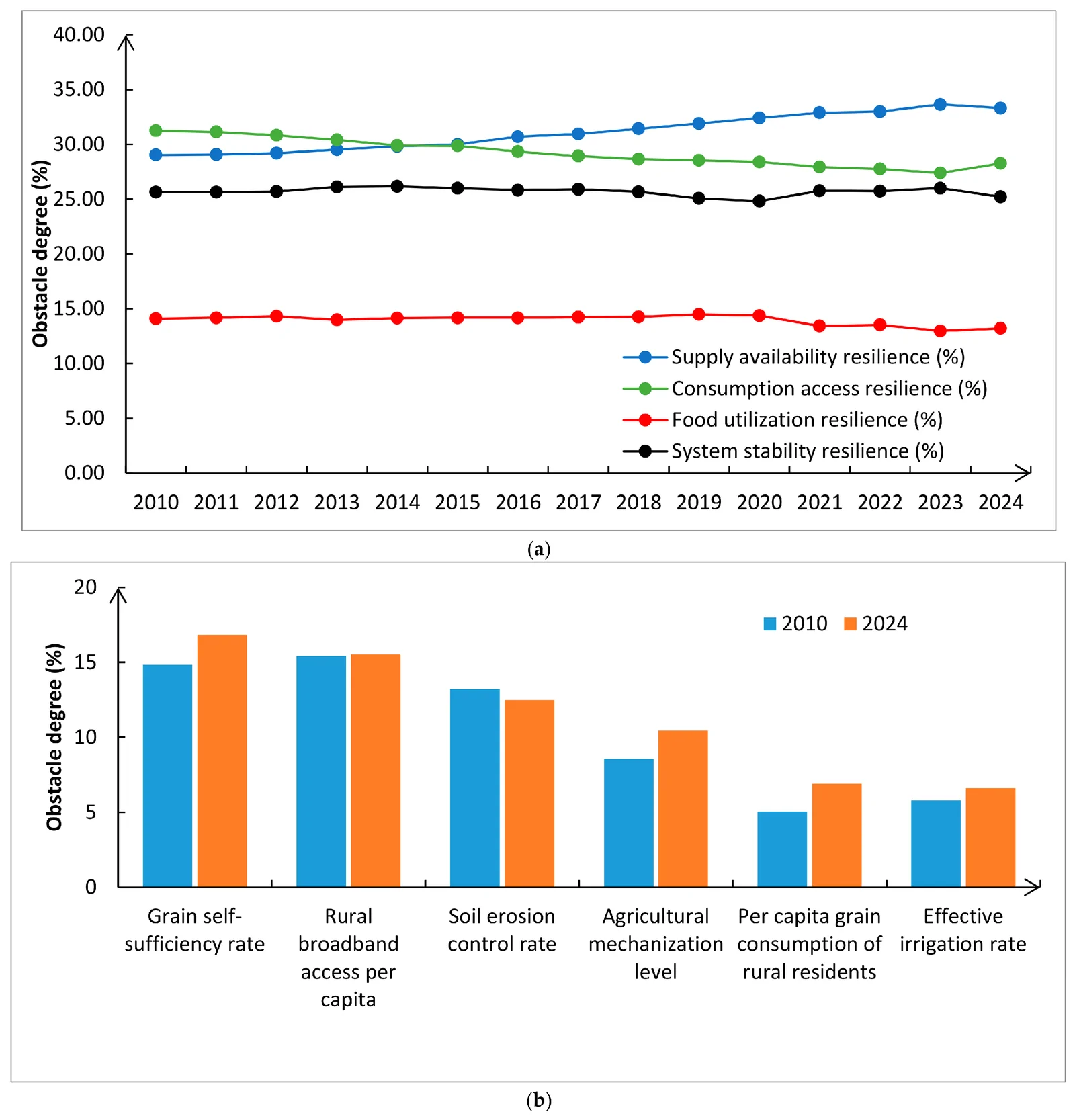
(**a**) Evolution of obstacle degrees of first-level indicators of China’s food security resilience, 2010–2024. (**b**) Obstacle degrees of the top six obstacle factors of China’s food security resilience.

The obstacle degree measurement results are shown in Figure 10. At the first-level indicator level (Figure 10a), the obstacle degree of supply availability resilience rose from 29.0% in 2010 to 33.3% in 2024, becoming the highest among the four dimensions, with the weighted shortfalls of the production and supply foundation and climate resistance capacity increasingly prominent; consumption access resilience edged down from 31.2% to 28.3%; system stability resilience remained broadly stable (25.6% and 25.2%); and food utilization resilience stayed the lowest (14.1% and 13.2%). Coordinating the supply defense line with the stability shield is therefore a priority area for resilience improvement in the context of climate change.

At the third-level indicator level (Figure 10b), the top five obstacle factors nationwide in 2010 were rural broadband access per capita (15.4%), the grain self-sufficiency rate (14.8%), the soil erosion control rate (13.2%), rural per capita disposable income (8.6%), and the agricultural mechanization level (8.6%); by 2024, they had evolved into the grain self-sufficiency rate (16.8%), rural broadband access per capita (15.5%), the soil erosion control rate (12.5%), the agricultural mechanization level (10.4%), and per capita grain consumption of rural residents (6.9%). The grain self-sufficiency rate has become the leading obstacle factor, rural broadband access and the soil erosion control rate have remained in the top three throughout, and the agricultural mechanization level has moved up markedly—pointing to persistent weighted shortfalls in the production capacity foundation, digital infrastructure, and ecological governance.

By region, the top five obstacle factors in 2024 are highly convergent in composition but differ in ranking (Table 4). The grain self-sufficiency rate leads in the eastern and central regions (21.2% and 17.4%, respectively), whereas rural broadband access per capita ranks first in the western and northeastern regions (15.3% and 16.6%) and within the top three in all four regions; the soil erosion control rate ranks among the top three in the northeastern (14.9%), western (14.6%), and central (13.0%) regions. Notably, the northeastern region’s list is further distinguished by the effective irrigation rate (10.2%) and road network density (8.2%), pointing to infrastructure-oriented improvement priorities, whereas the eastern region is more constrained by the production capacity foundation—calling for differentiated regional resilience improvement strategies.

From the perspective of climate exposure, the obstacle structures of the two exposure groups identified in Section 3.1 have clearly diverged by 2024. The weaknesses of the high-exposure group are concentrated in the production foundation—the grain self-sufficiency rate (17.7%) and the agricultural mechanization level (11.2%)—whereas its climate buffering indicators have improved faster: the obstacle degrees of the soil erosion control rate (11.1% vs. 13.9% in the low-exposure group) and the effective irrigation rate (6.1% vs. 7.2%) are both lower, with the inter-group gap in the former widening from about 0.005 in 2010 to about 0.028 in 2024; accordingly, the soil erosion control rate remains among the top three obstacles in the low-exposure group but not in the high-exposure group. This pattern corroborates the shock–response mechanism from the perspective of weakness diagnosis: frequently exposed provinces have preferentially reinforced the buffering links closest to climate shocks, so that their remaining bottlenecks have shifted toward the production and supply foundation, whereas less-exposed provinces still face unmet needs in ecological governance.

### 3.9. Potential Forecasting of China’s Food Security Resilience

The grey prediction GM(1,1) model is adopted to forecast food security resilience. Given the short sample span (2010–2024) and the smooth, monotonically increasing trajectory of the composite scores, the model is estimated for the nation and each province, with the development coefficient and grey action quantity obtained by least squares; all models pass the posterior-error and small-error-probability accuracy tests, with the RMSE of provincial fits ranging from 0.0042 to 0.0290. Rolling-window out-of-sample validation (a ten-year estimation window rolled forward one year at a time, generating one-step-ahead forecasts for 2020–2024 in 11 provinces selected by stratified sampling across the four major regions) yields MAE = 0.0159, RMSE = 0.0204, and MAPE = 4.68%, of the same order of magnitude as a naive persistence benchmark (MAE = 0.0133, RMSE = 0.0153, MAPE = 3.94%); GM(1,1) is adopted because naive methods cannot extrapolate over a five-year horizon. All forecasts are reported on their original scale without upper-bound truncation and should be interpreted as exploratory extrapolations of historical trends.

**Figure 11:**
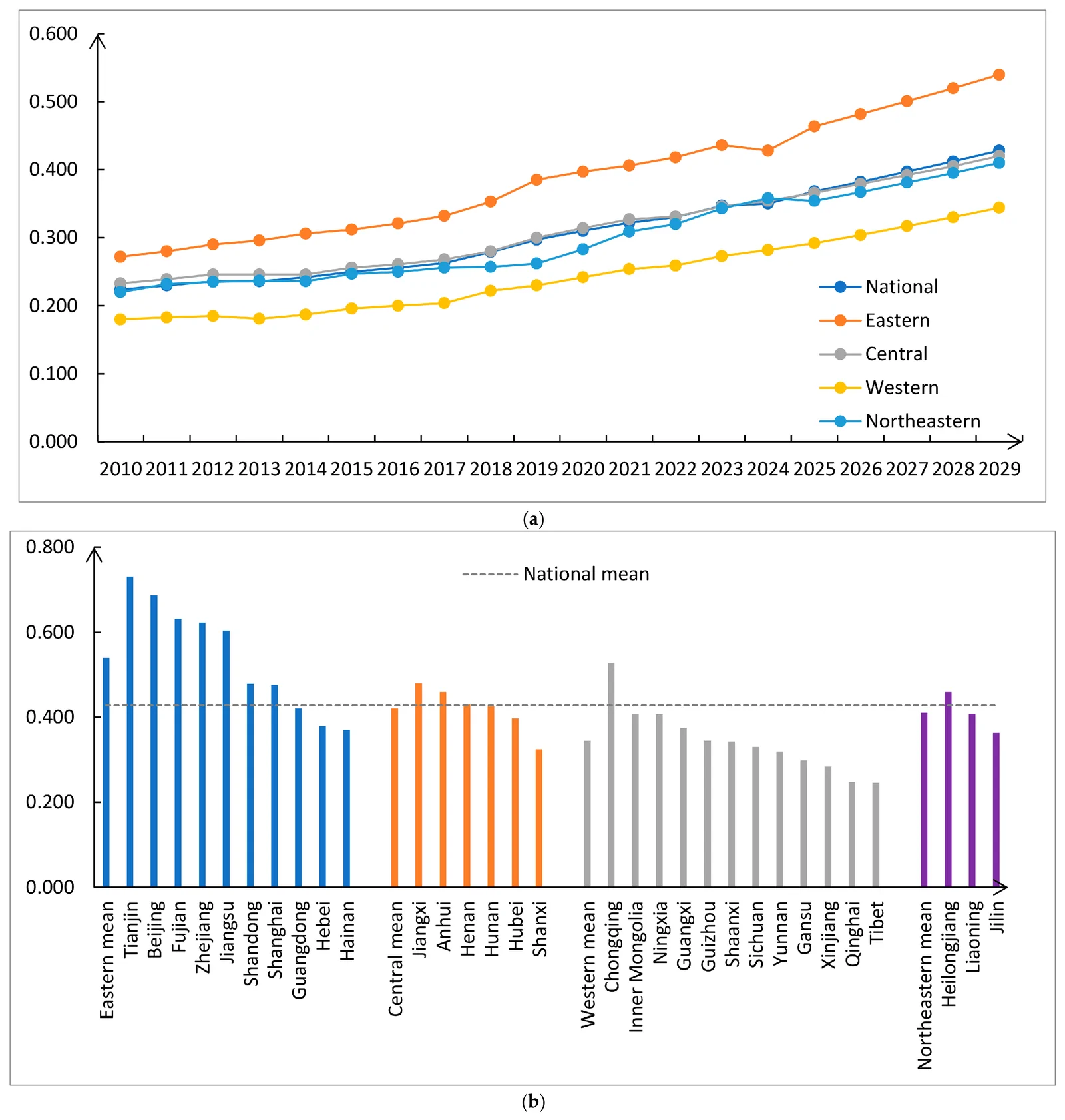

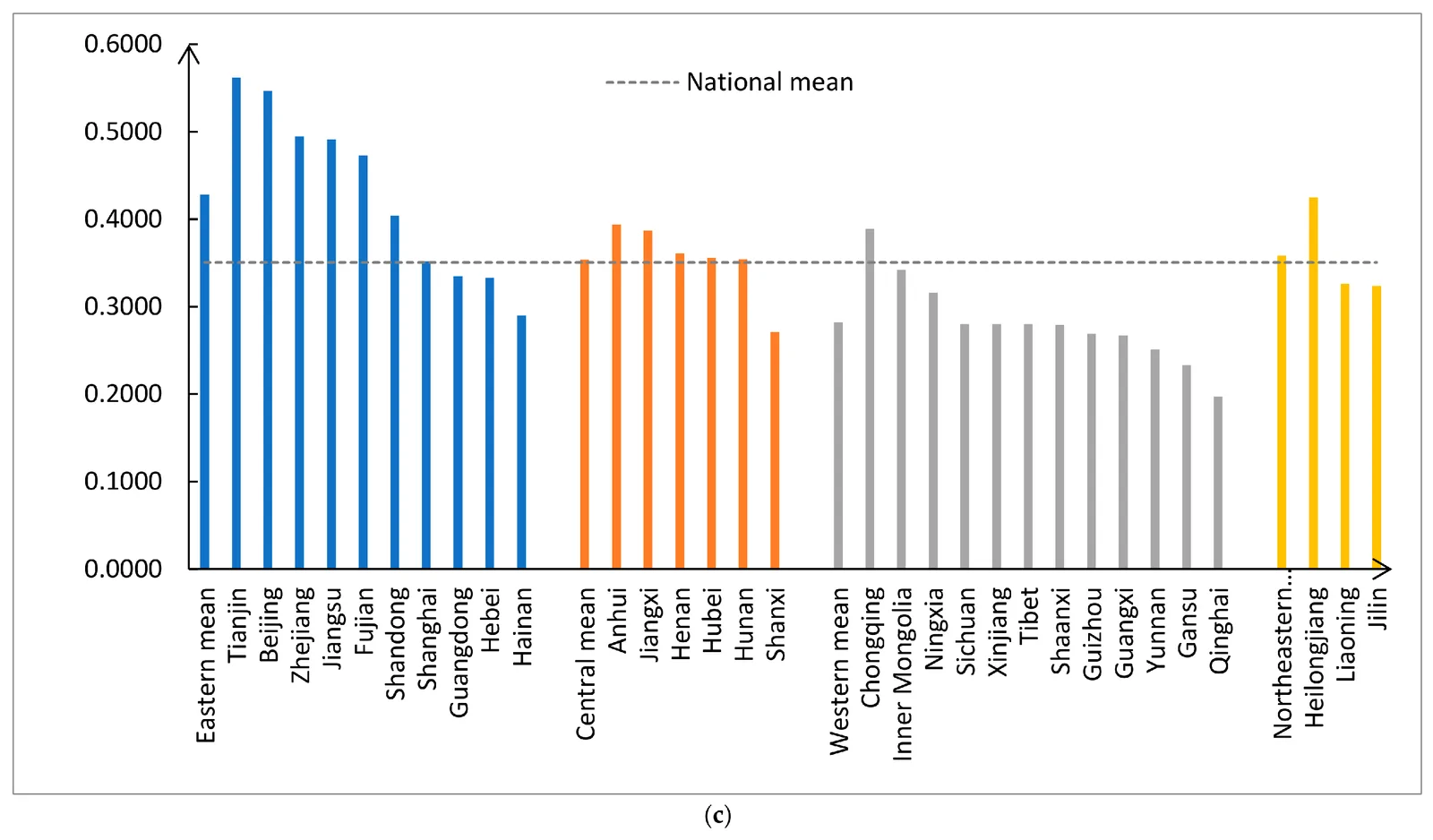
(**a**) GM(1,1) fitting and forecasting of food security resilience for the nation and the four major regions. (**b**) GM(1,1) forecast results of China’s food security resilience, 2029. (**c**) Composite scores of food security resilience for the nation and the four major regions, 2024.

Analysis of national and regional forecast results. As shown in Figure 11a, the national forecast value rises year by year from 0.368 in 2025 to 0.428 in 2029, extending the steady upward trend of 2010–2024. The gradient pattern among the four major regions remains stable: the eastern region rises from 0.464 to 0.540, consistently ranking first with a slowly widening lead; the central (0.366 to 0.420) and northeastern (0.354 to 0.410) regions move closely together; and the western region rises from 0.292 to 0.344, maintaining steady growth momentum from the lowest base.

Provincial forecasts and future scenario analysis. Based on the 2029 forecast values in Figure 11b, the provinces fall into three blocs: a leading bloc comprising Tianjin (0.731), Beijing (0.687), Fujian (0.632), Zhejiang (0.623), and Jiangsu (0.604); a steady catching-up bloc represented by Heilongjiang (0.460), Henan (0.430), Hunan (0.425), and Liaoning (0.408); and a low-position lagging bloc comprising Gansu (0.298), Yunnan (0.319), Xinjiang (0.284), and Qinghai (0.247). Tibet’s forecast value (0.246 in 2029) is even below its own 2024 level (0.280), making it the only one of the 31 provinces projected to decline rather than rise—a risk point demanding the most attention in future potential tapping. Compared with 2024, Tianjin (from 0.562 to 0.731), Fujian (from 0.473 to 0.632), Beijing (from 0.547 to 0.687), and Chongqing (from 0.389 to 0.528) register the largest increases. Overall, the eastern coastal provinces will continue to lead, while the western provinces, particularly those in the southwest and northwest, remain the key areas for potential tapping during the 15th Five-Year Plan period.

It must be clearly recognized that the TOPSIS relative closeness is a relative measure of the ideal state within the sample period, and that the forecasts extrapolate historical improvement trends under unchanged external conditions. If climate change-induced extreme events intensify beyond expectations and water and soil resource constraints tighten further, the supply and stability sides may drag down the overall upgrading process; conversely, if disaster prevention and mitigation, high-standard farmland construction, and risk-sharing mechanisms work in concert, provinces with large gaps from the leading bloc, such as Qinghai, Gansu, and Yunnan, are expected to accelerate their catching-up [38].

## 4. Discussion

Beyond the evolution patterns themselves, the results admit three deeper interpretations of how food security resilience is actually built under climate change. First, resilience gains appear to be driven mainly by the accumulation of general-purpose buffers rather than by food-specific instruments: the counterfactual decomposition attributes over 60% of the national improvement to ecological governance (soil erosion control, 24.7%), rural digital infrastructure (broadband access, 20.5%), and income growth (18.9%), whereas the contribution of the agricultural insurance loss ratio is negligible. This suggests that food security resilience has so far advanced largely as a by-product of broad rural modernization, while market-based risk-transfer mechanisms remain shallow and have yet to play a measurable buffering role. Second, adaptation appears to be reactive rather than anticipatory: provinces with higher mean flood exposure improved significantly faster than low-exposure provinces (0.1522 vs. 0.0988), indicating that adaptive investment—post-disaster reconstruction, irrigation and drainage upgrading, and insurance uptake—tends to follow losses rather than precede them; the significantly negative coefficients of the flood-affected rate and temperature variability in the panel analysis further confirm that realized climate shocks erode the composite score, so that resilience must be continuously rebuilt under repeated stress rather than accumulated once and for all. Third, the strong state dependence revealed by the Markov analysis (the lowest and highest quartiles persist with probabilities of 0.84 and 0.98, respectively) implies that initial conditions and neighborhood context lock provinces into distinct trajectories: latecomer provinces are unlikely to converge autonomously and will require sustained external support to escape the low-resilience club. These interpretations also clarify the real-world meaning of the forecasts to 2029: the projected persistence of regional gaps reflects the compounding advantage of accumulated buffers in the leading bloc rather than any deterioration in lagging regions, and the gaps between individual provinces and the ideal frontier identify where future improvement potential is concentrated.

The findings of this study exhibit both convergence with and divergence from the existing literature. In terms of convergence, the overall upward trend in resilience levels is consistent with the findings of Zuo and Ye [9] and Li et al. [8] on food system and agricultural climate resilience, and with the measurement of China’s food security resilience under a climate change framework by Xie et al. [13]; the spatial differentiation observed by Yang et al. [12], Yin et al. [10], and Lin et al. [21] also resembles the pattern identified here, and the obstacle diagnoses of Yang and Chen [17] and Han et al. [18] likewise identify weaknesses among infrastructure and risk protection factors. Such convergence, however, cannot by itself be taken as validation of the composite index: because these studies employ similar indicator families and measurement approaches, agreement in conclusions is to be expected and does not constitute independent evidence of validity. A more meaningful source of external validation is the panel econometric analysis in Section 3.1, in which realized climate shocks—the flood-affected rate and temperature variability—significantly reduce the composite score; the index thus responds to observed climatic stressors in the direction predicted by resilience theory, providing theory-consistent evidence that the measure captures exposure to climate shocks rather than merely reflecting secular development trends. This is consistent with the emphasis of Zhao et al. [31], Chen and Hu [39], and Su et al. [38] on the impact of climate change on the stability of grain supply. Divergence is equally informative. Whereas existing measurement studies mostly stop at level measurement or disparity decomposition, the exposure-grouping analysis of this study reveals a shock–response pattern—high-exposure provinces improved their resilience significantly faster than low-exposure ones—that has rarely been documented; moreover, the forward-looking forecasting extends the analysis to 2029, an extension that existing studies rarely attempt.

Relative to the existing literature, this study differs mainly in three respects. First, its evaluation framework fits the climate change scenario more closely. Whereas existing measurement studies have mostly constructed indicators from the single perspective of supply security, industrial systems, or the agricultural economy [8,9,11], this study, grounded in the context of climate change, constructs an evaluation system for food security resilience covering the four dimensions of supply availability, consumption access, food utilization, and system stability, and explicitly incorporates climate adaptation and risk protection indicators such as the effective irrigation rate, the soil erosion control rate, and the agricultural insurance loss ratio, so that the measurement results better characterize the system’s response capacity under climate shocks. Second, its method chain is more complete. Whereas existing studies mostly stop at a single step such as level measurement or regional disparity decomposition [10,12,21], this study integrates entropy–TOPSIS measurement, Dagum Gini coefficient decomposition, kernel density estimation, the spatial Markov chain, the obstacle degree model, and GM(1,1) grey forecasting into a unified framework, forming a complete, interlocking analytical chain of “level measurement–disparity decomposition–spatiotemporal evolution–obstacle diagnosis–potential forecasting”, and characterizes the “club convergence” of resilience-level transitions and the association between neighborhood states and transition patterns. Third, its research perspective is more forward-looking. Forecasting studies targeting food security resilience are rare in the existing literature; based on the GM(1,1) grey prediction model, this study extends the analysis to 2029 and quantitatively assesses the gaps between individual provinces and the ideal frontier as well as their improvement potential, making up for the lack of forward-looking evidence in this field.

The findings of this study carry implications for policy, within the limits of what the analysis can support. The obstacle diagnosis identifies weighted shortfalls relative to the sample frontier rather than causal constraints; accordingly, the persistent leading positions of the grain self-sufficiency rate, rural broadband access per capita, and the soil erosion control rate suggest that consolidating the production capacity foundation, rural digital infrastructure, and ecological governance are plausible priority areas, but they do not identify the causal effects of any specific policy instrument—such as agricultural insurance, digital infrastructure investment, or ecological programs—nor an optimal allocation of public resources. In a similarly descriptive vein, the regional heterogeneity of obstacle structures and the exploratory forecasts to 2029 may serve as reference points for regionally differentiated attention—for example, monitoring provinces projected to lag or decline, such as Qinghai, Gansu, Yunnan, and Tibet—rather than as a prescriptive allocation scheme.

Although this study investigates the level measurement, spatiotemporal evolution, obstacle diagnosis, and potential forecasting of food security resilience in the context of climate change from a provincial macro perspective, it still has certain limitations. (1) At the data level, the study relies mainly on various statistical yearbooks; the statistical calibers of indicators such as agricultural insurance and soil erosion control differ slightly across provinces, and individual missing observations are filled by linear interpolation or nearest-neighbor extension, so minor measurement errors are unavoidable. (2) In terms of research scale, this study stops at the provincial macro level and does not descend to the prefecture-city and county scales, nor does it conduct heterogeneity analyses for different grain varieties such as wheat and rice or for different types of climate disasters such as droughts and floods. (3) In terms of mechanism identification, although the panel econometric analysis verifies the aggregate effects of climate shocks, the micro-level transmission channels through which agricultural insurance, disaster prevention and mitigation investment, and other factors shape resilience are not formally tested, and the Markov transition probabilities are descriptive in nature. (4) The measurement results depend on the composition of the indicator set and on the entropy weighting scheme: although the equal-weight and Top-3 stress tests preserve the provincial rankings (Pearson r = 0.872–0.966), a fuller sensitivity analysis of indicator inclusion and exclusion remains for future work; likewise, the obstacle degree diagnosis identifies weighted shortfalls relative to the sample ideal solution rather than causal constraints, and its ranking partly reflects the entropy weights of indicators. (5) The spatial Markov analysis is conditional on the adjacency-based neighborhood definition; alternative spatial weight matrices (e.g., geographic-distance or economic-distance matrices) may yield different conditional transition patterns, and because neighboring provinces’ resilience is itself endogenous to common regional shocks and policy diffusion, the association between neighborhood states and transition probabilities should not be interpreted causally. (6) Several indicators are expressed in per-capita terms, which may favor provinces with smaller or declining populations and may understate the absolute supply capacity of populous provinces. (7) The GM(1,1) forecasts extrapolate stable grey tendencies and cannot anticipate structural breaks, major policy shifts, or unprecedented climate extremes; the projections to 2029 should therefore be read as exploratory baselines rather than precise predictions.

Future research can be deepened in the following directions. First, data sources can be expanded by integrating statistical materials at the prefecture, city, and county levels, micro-level agricultural insurance data, and multi-source information from meteorological stations and satellite remote sensing, cross-validating abnormal years, extending the research scale down to the county level, and improving the precision of indicator measurement. Second, the crop variety and disaster type dimensions can be refined by constructing classified evaluation frameworks for major grain varieties such as wheat, rice, and maize and for typical climate disasters such as droughts and floods, revealing heterogeneous patterns of resilience evolution. Third, econometric methods such as the spatial Durbin model, mediation effect models, and alternative spatial weight matrices can be introduced to empirically test the influence paths, transmission mechanisms, and constraint conditions of external factors—such as climate shocks, the expansion of agricultural insurance coverage, and the development of disaster prevention and mitigation systems—on food security resilience, thereby making up for the shortcomings of the current mechanism analysis.

## 5. Conclusions

First, measured by the composite index constructed in this study, from 2010 to 2024, the composite score of China’s food security resilience rose steadily from 0.2240 to 0.3504, with an average annual growth rate of 3.2475%, exhibiting a smooth and continuous upward trend without abnormal fluctuations. The entropy weights are relatively balanced across the four first-level dimensions, with consumption access (28.86%), supply availability (28.64%), and system stability (28.32%) carrying nearly equal weights, and the developed eastern provinces and municipalities rank at the forefront.

Second, food security resilience exhibits a significant and strong positive spatial autocorrelation throughout the study period, with the global Moran’s I index ranging between 0.4280 and 0.6390 and remaining significant at the 1% level in all years; the index rose from 0.4280 in 2010 to a peak of 0.6390 in 2019 and stood at 0.5290 in 2024. Moran scatterplots show that the “H-H” and “L-L” agglomeration patterns have clearly taken shape, and the spatial pattern is one of “high values along the eastern coast and relatively low values in the western and some central provinces”.

Third, the overall Gini coefficient fluctuated within the range of 0.140–0.162 and declined overall to 0.140 in 2024, with inter-group differences being by far the dominant source of disparity (average contribution rate of 70.13%). The center of gravity of the kernel density curve shifts rightward with widening dispersion. Transitions among resilience levels are characterized by “club convergence”, with the state-retention probability of the high-level club reaching 0.9798. In descriptive terms, neighborhood resilience levels are strongly associated with local transitions: an upgrading neighborhood environment is accompanied by a markedly higher probability of upward transition—the probability of medium-high-level provinces entering the high level rises from 4.00% under medium-low-level neighborhoods to 36.36% under high-level neighborhoods—whereas low-level neighborhoods are associated with a downward drag on adjacent provinces; given the limited number of observations in some cells, these conditional probabilities should be interpreted as indicative patterns rather than statistically tested effects.

Fourth, the grain self-sufficiency rate, rural broadband access per capita, and the soil erosion control rate are the top three obstacle factors nationwide (16.8%, 15.5%, and 12.5% in 2024, respectively). At the first-level dimension level, the obstacle degree of supply availability resilience rose from 29.0% to 33.3% and ranked highest by 2024, indicating a widening weighted shortfall of the production and supply foundation; while food utilization resilience remained the least obstructed dimension. By region, the leading obstacle is the grain self-sufficiency rate in the eastern and central regions and rural broadband access per capita in the western and northeastern regions, indicating a certain degree of regional heterogeneity in the obstacle structure.

Fifth, GM(1,1) grey prediction shows that the national food security resilience forecast value will rise from 0.368 in 2025 to 0.428 in 2029, with the eastern region maintaining its lead. Tianjin, Beijing, Fujian, Zhejiang, and Jiangsu will remain in the leading bloc; Tianjin, Fujian, Beijing, and Chongqing will register the largest increases; and Qinghai, Xinjiang, Gansu, and Yunnan will have the largest gaps from the national average. Among them, Tibet’s forecast value is below its 2024 level, making it the only province projected to decline rather than rise and a key area for potential tapping during the 15th Five-Year Plan period.

Sixth, the panel econometric results show that climate change exerts a significant negative driving effect on food security resilience: in the baseline two-way fixed effects model, the coefficients of the flood-affected rate and temperature variability are both negative (−0.045, *p* < 0.05; −0.022, *p* = 0.071), and the negative effects are significantly reinforced in the random effects, winsorized, and logit robustness specifications, providing direct econometric evidence for the “in the context of climate change” premise of this study. Moreover, the exposure-based grouping shows that provinces with high flood exposure improved their resilience significantly faster than low-exposure provinces (0.1522 vs. 0.0988, *p* = 0.013), corroborating a shock-driven pattern of adaptive capacity building.

## Figures and Tables

**Table 1:** Evaluation indicator system and weights of China’s food security resilience in the context of climate change.

First-Level Indicator (Weight %)	Second-Level Indicator (Weight %)	Third-Level Indicator	Definition	Direction	Weight
Supply Availability Resilience	Production & supply capacity	Grain yield per unit area [34]	Ratio of total grain output to grain sown area in the current year	Positive	3.00%
		Grain self-sufficiency rate [35]	Ratio of grain output to grain consumption	Positive	12.78%
	Climate resistance capacity	Multiple cropping index [8]	Ratio of total sown area of crops to cultivated land area	Positive	4.92%
		Agricultural mechanization level [36]	Ratio of total power of agricultural machinery to total sown area of crops	Positive	7.94%
Consumption Access Resilience	Economic accessibility	Per capita disposable income of rural residents [8]	Per capita disposable income of rural residents in the current year	Positive	7.17%
		Engel’s coefficient of rural residents [34]	Share of food, tobacco and alcohol expenditure in total consumption expenditure of rural residents	Negative	1.82%
	Market circulation capacity	Road network density [31]	Ratio of highway mileage to administrative area	Positive	6.99%
		Rural broadband access per capita [37]	Ratio of rural broadband subscribers to rural resident population	Positive	12.88%
Food Utilization Resilience	Nutritional outcomes	Per capita grain consumption of rural residents [27]	Per capita consumption of staple grains by rural residents; measures the quantity security of staple food intake—the foundation of the dietary structure—rather than the overall nutritional quality	Positive	4.90%
		Consumption of high-quality protein foods [31]	Per capita consumption of meat, eggs, milk and aquatic products by rural residents; the share of high-quality protein foods in the diet is a standard proxy for dietary quality upgrading, widely used in the literature	Positive	5.09%
	Safe utilization conditions	Rural water supply coverage rate [38]	Ratio of population benefiting from centralized water supply to rural population	Positive	0.44%
		Health technicians per 10,000 population [4]	Ratio of health technicians to resident population (per 10,000 persons)	Positive	3.76%
System Stability Resilience	Climate disaster buffering	Soil erosion control rate [36]	Ratio of controlled soil erosion area to total soil erosion area	Positive	12.32%
		Effective irrigation rate [8]	Ratio of effectively irrigated area to total cultivated land area	Positive	7.49%
	Risk sharing & regulation	Agricultural insurance loss ratio [25]	Ratio of agricultural insurance claim payments to premium income in the current year; measures the realized payout pressure on the agricultural insurance system under disaster shocks (higher values indicate greater disaster losses)	Negative	0.76%
		Intensity of fiscal support for agriculture [31]	Ratio of expenditure on agriculture, forestry and water affairs to general public budget expenditure	Positive	4.11%
		Rural pension insurance participation rate [25]	Ratio of participants in basic old-age insurance for urban and rural residents to rural population	Positive	3.64%

**Table 2:** Pearson correlations among food security resilience scores under alternative weighting schemes.

Score Series	Baseline	Equal Weights	Perturbed Weights
Baseline	1		
Equal weights	0.908 **	1	
Perturbed weights	0.872 **	0.966 **	1

Notes: ** *p* < 0.01; N = 465. Baseline refers to the entropy-weighted TOPSIS scores; equal weights assigns 1/17 to each indicator; the Top-3 stress test scales the weights of the three highest-weighted indicators by 0.2 and redistributes the released weight equally among the remaining 14 indicators.

**Table 3:** Panel regression results of climate driving effects on food security resilience.

Variable	Two-Way FE	RE	RE (Winsor.)	RE (Logit)
flood-rate	−0.045 *	−0.114 **	−0.141 **	−0.508 **
	(−2.227)	(−2.654)	(−3.048)	(−2.989)
temp-var	−0.022	−0.087 **	−0.088 **	−0.377 **
	(−1.812)	(−3.678)	(−3.702)	(−3.736)
precip-var	−0.005	−0.001	−0.001	−0.004
	(−1.059)	(−0.234)	(−0.200)	(−0.181)
urban	−0.369 **	0.479 **	0.475 **	2.714 **
	(−2.839)	(9.230)	(9.783)	(9.862)
grain-sow-ratio	0.116 **	0.111 **	0.110 **	0.516 **
	(5.255)	(3.860)	(4.077)	(4.070)
primary ratio	0.001	−0.001	−0.001	−0.002
	(0.297)	(−1.257)	(−1.000)	(−0.252)
pop-density	−0.006	−0.008	−0.006	−0.043
	(−0.051)	(−0.586)	(−0.495)	(−0.605)
Constant	0.434	−0.013	−0.019	−2.650 **
	(0.747)	(−0.154)	(−0.240)	(−5.328)
R^2^ (within)	0.399	0.692	0.705	0.738
N	465	465	465	465

Notes: *t*-values in parentheses; * *p* < 0.05, ** *p* < 0.01; cluster-robust standard errors at the provincial level; precip-var is scaled by 100; pop-density is logarithmically transformed(ln); the winsorized specification re-estimates the RE model after winsorizing all continuous variables at the 1st and 99th percentiles; the logit specification uses ln(C/(1−C)) as the dependent variable. Model selection: F test F(30,427) = 46.179 (*p* < 0.01), BP test χ^2^(1) = 1681.311 (*p* < 0.01), Hausman test χ^2^(7) = 0.930 (*p* = 0.996).

**Table 4:** Five obstacle factors and their obstacle degrees by region in China, 2024.

Rank	Eastern		Central		Western		Northeastern	
	Obstacle factor	Obstacle degree (%)	Obstacle factor	Obstacle degree (%)	Obstacle factor	Obstacle degree (%)	Obstacle factor	Obstacle degree (%)
1	Grain self-sufficiency rate	21.22	Grain self-sufficiency rate	17.37	Rural broadband access per capita	15.26	Rural broadband access per capita	16.59
2	Rural broadband access per capita	14.53	Rural broadband access per capita	17.24	Grain self-sufficiency rate	15.19	Soil erosion control rate	14.91
3	Agricultural mechanization level	11.54	Soil erosion control rate	12.98	Soil erosion control rate	14.64	Agricultural mechanization level	10.48
4	Soil erosion control rate	8.85	Agricultural mechanization level	10.92	Agricultural mechanization level	9.27	Effective irrigation rate	10.22
5	Per capita grain consumption of rural residents	8.09	Per capita grain consumption of rural residents	6.97	Effective irrigation rate	7.76	Road network density	8.19

## Data Availability

The original contributions presented in this study are included in the article. Further inquiries can be directed to the corresponding author.
